# From court to community: a cost–benefit evaluation of a community sports programme for Arab women in Israel's multiethnic context

**DOI:** 10.3389/fgwh.2025.1661221

**Published:** 2025-12-11

**Authors:** Limor Dina Gonen, Sharon Barak, Karin Eines, Ruth Birk, Riki Tesler

**Affiliations:** 1 Department of Economics and Business Administration, Ariel University, Israel; 2 Department of Nursing, Faculty of Health Sciences, Ariel University, Israel; 3 Department of Health Systems Management, Faculty of Health Sciences, Ariel University, Israel; 4 Department of Nutrition, Faculty of Health Sciences, Ariel University, Israel

**Keywords:** Mamanet, community sports, women's health, social capital, cultural adaptation, empowerment programmes, cost-benefit analysis (CBA), quality-adjusted life years

## Abstract

**Background:**

Physical activity (PA) is a key determinant of women's physical, mental, and social well-being, yet participation in structured sports remains limited due to sociocultural norms, caregiving roles, and accessibility barriers. The Mamanet Cachibol League (Mamanet), a community-based sports initiative in Israel, addresses these barriers by fostering participation among mothers from diverse backgrounds. This study evaluates Mamanet's health and economic impacts among Arab women through a cost–benefit analysis (CBA) and quality-adjusted life year (QALY) framework.

**Methods:**

A quantitative pre–post evaluation was conducted with 174 Arab women participating in Mamanet teams across diverse geographic and socioeconomic contexts in Israel. The non-randomized design examined within-participant changes over a 10-month period, providing real-world evidence of programme effectiveness. Participants completed validated Hebrew and Arabic questionnaires before and after the intervention. Health-related variables included self-rated health, psychosomatic symptoms, physical activity, mental well-being, and social capital indicators. The economic evaluation incorporated reductions in healthcare utilization and medication costs, productivity gains, and QALY improvements, accounting for direct and opportunity costs.

**Results:**

Mamanet participation was associated with improvements in physical health outcomes, including a significant reduction in psychosomatic symptoms (*p* < 0.001, effect size = 0.75). Gains in mental well-being were observed but did not reach statistical significance (*p* = 0.09). Modest increases were found in social capital and community engagement. Economically, participation in the programme was associated with reduced healthcare use, lower absenteeism, and decreased medication expenditures, as well as enhanced productivity. The benefit–cost ratio (BCR) was 1.13, indicating that overall benefits exceeded programme costs.

**Conclusion:**

The Mamanet programme yields measurable health, social, and economic benefits for Arab women in Israel, demonstrating how culturally adapted, community-based interventions can reduce barriers to physical activity and promote health equity. Its low-cost, inclusive model offers a scalable framework for integrating women's sports into public health strategies and strengthening community resilience. Beyond Israel, the programme's principles affordability, inclusion, and engagement can inform policies supporting the Sustainable Development Goals (SDG), particularly those addressing gender equality, well-being, and reduced inequalities.

## Introduction

Physical activity (PA) is defined as any bodily movement produced by skeletal muscles requiring energy expenditure (1). Although there are many benefits of adopting PA, women's participation in PA continues to be lower than that of men, thereby sustaining gender-based health inequities (2, 3). Women, particularly in marginalized communities, are disproportionately affected by sociocultural expectations, limited access to facilities, financial restrictions, and time obligations, frequently linked to caregiving responsibilities (2, 3). A promising strategy to enhance women's PA participation, is the implementation of community-based programmes targeted at specific populations, offering a supportive and culturally sensitive environment to address common barriers to PA, particularly for women from cultural backgrounds with traditional gender role expectations (4). Moreover, community sports initiatives strengthen social capital, empowerment, and integration. By participating in team-based activities, women create networks of mutual support and accountability that can improve self-confidence and leadership abilities (5, 6). Participation in club-based or team sports has been linked to improved psychological and social well-being, such as enhanced self-esteem and emotional resilience (2, 3). For example, Brewer et al. (7) illustrated how the FAITH! project improved cardiometabolic and psychological health in African American women.

Community-based interventions (CBIs) yield considerable economic and societal impacts, making them relevant to policymakers and public health strategists. Women's participation in team sports is associated with a reduction in health complications, leading to lower healthcare costs and decreased pressure on public health systems (2, 8). Enhanced fitness similarly correlates with higher workplace productivity, reduced absenteeism, and even improved career prospects, especially among women juggling caregiving with employment (2, 9). On a broader societal level, well-executed sports initiatives foster social capital, generate public health benefits, and stimulate communal cohesion (8). The interconnected results have led researchers to use the methodology of cost-benefit analysis (CBA) and quality adjusted life years (QALYs) to measure long-term benefits of public or private investment in community-based sports programmes (10). The benefit-cost ratio (BCR) is essential for assessing a programme's feasibility from an economic perspective. Policymakers frequently utilize these metrics to identify high-impact, cost-effective interventions (8, 10). The integration of QALYs in health-related analysis provides an additional measure that encompasses both the duration and quality of participants' lives (11).

Established in 2005, the Mothers Cachibol League (Mamanet) is the largest model of community engagement in a social-sports league specifically for mothers in Israel today. With thousands of mother-players in over 90 cities across Israel, Mamanet is now spreading to countries such as Austria, Italy, Greece, Cyprus, and the U.S (5). Initial findings indicate significant improvements in participants' physical well-being, enhanced mental health, and social capital, alongside a potential reduction in healthcare costs (8, 12). The programme's focus on team-building and local collaboration suggests significant productivity advantages, frequently indicated by participants' reduced work absences and increased confidence in professional environments (13, 14).

Conventional sports economics literature primarily has focused on male-dominated sectors or broad population-level initiatives (6, 9). This study aims to address the limited attention paid to women-specific sports interventions, particularly those targeting women from cultural backgrounds with lower PA levels and greater barriers to participation. The outcomes of women's community-based team sports participation are evaluated by conducting a comprehensive cost-benefit analysis of Mamanet.

### Key research questions

Based on the programme's conceptual framework and the existing empirical literature on community-based physical activity and women's health promotion, the present study addressed the following open-ended research questions.

These questions were formulated to explore the *extent* and *nature* of associations between participation in the Mamanet programme and multiple dimensions of health and well-being, without assuming the direction or magnitude of effects.

1. To what extent does participation in the Mamanet programme influence participants' self-perceived physical health?

This question investigates the relationship between engagement in structured, community-based physical activity and women's perceived general health, consistent with evidence that participatory sports interventions are associated with improved physical functioning and vitality (3, 15).

2. In what ways does Mamanet participation relate to changes in psychosomatic symptoms among participants?

This question assesses whether participation in a supportive sport environment corresponds to lower stress-related somatic complaints, as suggested by prior research on exercise and psychosomatic health among women (16).

3. How does participation in Mamanet influence participants' mental well-being and emotional balance?

This question focuses on subjective mental health outcomes such as mood, self-esteem, and stress regulation, given growing evidence linking recreational sport participation to psychological resilience and happiness (15).

4. What relationship exists between participation in Mamanet and levels of social capital and community engagement?

This question explores the programme's potential role in fostering social cohesion, trust, and mutual support among women, aligning with the literature on sports as a vehicle for community-building and intergroup contact (17, 18).

5. What is the effect of Mamanet participation on participants' healthcare utilization and related economic outcomes?

This final question extends the analysis into the economic domain, assessing potential associations with healthcare use, absenteeism, and productivity, consistent with cost–benefit and cost–utility frameworks in health economics (19, 20).

Together, these open-ended research questions provide a comprehensive framework for examining how structured, community-based sport participation may contribute to physical, psychological, social, and economic well-being among women from diverse sociocultural backgrounds in Israel.

The study's innovative research perspective integrates both health and economic frameworks to evaluate women's participation in community-based team sports, atypically examined from a purely public health standpoint. CBA and QALYs are combined with conventional health outcomes to yield substantial quantitative evidence regarding the measurable benefits of programmes such as Mamanet for individuals as well as society. Given the programme's demonstrated success within Israel's multicultural society, Mamanet may serve as a replicable model for promoting women's health and community cohesion in other multicultural or underserved populations worldwide. Its focus on inclusivity, social capital, and affordability makes it particularly relevant to low- and middle-income countries aligning with global efforts to achieve SDG 3 (Good Health and Well-being), SDG 5 (Gender Equality), and SDG 10 (Reduced Inequalities).

## Background

### Literature review

Physical activity (PA) is a key determinant of women's physical, mental, and social well-being. Regular participation contributes to reduced morbidity, improved functional capacity, and enhanced emotional stability across the lifespan. Yet, despite these benefits, women particularly those from minority and low-income backgrounds remain less engaged in organized physical activity due to sociocultural norms, caregiving responsibilities, and limited access to affordable opportunities (21, 22).

The social and emotional competencies acquired via athletics, including teamwork, leadership, and dispute resolution, are skills that enhance personal connections and facilitate professional achievement. Engagement in team sports provides an avenue for empowerment by fostering self-efficacy, resilience, and cooperative learning, thereby strengthening both individual and collective capabilities (23).

Israel provides a unique sociocultural setting for evaluating community-based health and sport interventions. The population is highly diverse, comprising Jewish, Arab, Druze, and other minority groups that differ in cultural norms, socioeconomic conditions, and health behaviors. This diversity exists alongside historical and political tensions that often constrain intergroup contact and shared civic participation (24). In this context, the Mamanet programme functions not only as a health-promotion initiative but also as a structured environment for intergroup engagement and social inclusion. The league's organization of mixed-ethnicity tournaments, bilingual communication (Hebrew and Arabic), and family-oriented community events enables meaningful contact across cultural boundaries. These elements align with intergroup contact theory (25), which posits that sustained cooperation under equal-status conditions fosters mutual understanding and reduces prejudice. Consistent with recent empirical evidence from community-based sport programmes in divided societies (18, 25), such encounters can strengthen social capital by enhancing trust, reciprocity, and shared identity. Thus, beyond its physical and psychosocial health benefits, Mamanet represents a culturally adapted mechanism that may foster social cohesion, well-being, and collective resilience within a diverse and sometimes polarized society.

Team sports offer psychological and social advantages for underprivileged populations by fostering an inclusive atmosphere where individuals from diverse backgrounds unite around shared objectives rather than social disparities. Such initiatives enhance self-esteem and reduce psychological distress among marginalized groups (15, 16).

Team-based sports also play a pivotal role in the development of social capital, creating enduring networks of trust, reciprocity, and mutual accountability that extend beyond the sports arena and contribute to community resilience (26, 27). This collaborative behavior promotes a culture of assistance and civic engagement, reinforcing social cohesion and collective efficacy (28, 29). Programmes like Mamanet demonstrate that diversity-oriented sports can effectively unite women from different cultural, social, and religious backgrounds, facilitating the dismantling of stereotypes, reducing prejudice, and promoting social integration (18, 25). Elevated levels of community social capital are strongly associated with better access to resources, healthier behavioral patterns, and greater perceived safety within the community, establishing a virtuous cycle of engagement and well-being.

The substantial physical, mental, and social advantages of team sports have led to increasing advocacy for their integration into public health policy. Governments and health organizations are encouraged to allocate resources to community-based sports initiatives that enhance participation and equity (21, 28). Greater engagement in team sports can markedly reduce chronic disease prevalence and associated healthcare costs (19, 20). Community programmes targeting women, youth, and at-risk populations can foster a healthier, more productive society that requires fewer medical interventions (22).

Collaboration among the health, education, and sports sectors is essential for sustaining the long-term benefits of community-based physical activity programmes. Integrating team sports into school curricula can promote lifelong engagement in physical activity, while healthcare providers may reinforce these behaviours through preventive or rehabilitative exercise prescriptions. Such intersectoral partnerships create the systemic foundation needed to scale inclusive and culturally adapted initiatives such as the Mamanet programme ensuring that sport-based health promotion is embedded within broader public health policy frameworks and remains accessible to diverse demographic groups, including older adults and women balancing caregiving and professional duties.

### Community-based interventions as vehicles for health promotion—the Mamanet model

Mamanet, derived from the international game of Cachibol (a modified volleyball-based team sport), was established in Israel in 2005 with the explicit aim of encouraging mothers' participation in organized physical activity. The game involves six players per team and is played on a standard volleyball court with moderate-intensity aerobic effort combining movement, coordination, and teamwork. Beyond its physical component, Mamanet's structured weekly sessions and community-based teams promote social interaction, inclusion, and well-being among women from diverse cultural and socioeconomic backgrounds.

Mamanet now operates across more than 100 municipalities in Israel, integrating Jewish and Arab women through mixed tournaments, bilingual communication, and family-oriented community events. This inclusive design reflects a culturally sensitive adaptation of global team-sport models to local realities. As a result, Mamanet functions not only as a health-promotion initiative but also as a platform for intergroup contact, social trust, and empowerment consistent with intergroup contact theory (30).

Community-based physical activity programmes like Mamanet serve as effective CBIs by addressing barriers to participation, particularly those related to caregiving, affordability, and cultural constraints. Such initiatives embed health promotion within existing community networks, providing accessible, low-cost, and socially supportive environments (15, 27).

From an economic perspective, Mamanet demonstrates how localized, inclusive sports programmes can enhance productivity, reduce healthcare utilization, and improve cost-effectiveness outcomes. These findings illustrate the dual role of CBIs in fostering both population health and social capital, offering a scalable model for equitable public health promotion (18, 26).

Preventive health interventions are increasingly recognized for their capacity to generate long-term economic benefits by reducing healthcare expenditures, improving workforce productivity, and enhancing population well-being. These interventions, particularly when implemented through community-based platforms, offer governments an effective mechanism for alleviating the financial burden of non-communicable diseases (NCDs) while improving QALYs and social cohesion.

CBA, cost-effectiveness analysis (CEA), and cost-utility analysis (CUA) are essential tools for evaluating health programmes. CBA compares implementation costs with benefits such as reduced healthcare usage and enhanced productivity (31). The CEA focuses on cost per health outcome unit (e.g., life-years saved), while CUA incorporates QALYs, combining quantity and quality of life (32, 33).

Productivity gains are central to the economic case for women's participation in team sports. By reducing absenteeism and improving energy, confidence, and mental clarity, participation contributes to better workplace performance (14). These benefits are especially important among women balancing caregiving and employment, where stress and fatigue often undermine output and advancement. From a macroeconomic perspective, the impact of such programmes extends to healthcare budgets, gross domestic product (GDP) contributions, and intergenerational labour outcomes (34). Increased workforce participation among women who would otherwise be excluded or marginalized due to health or social isolation contributes directly to economic growth. When modelled using labour market simulations, team-sport interventions improve projected human capital indices and life satisfaction scores, indicators closely tied to national well-being (35). The estimated cost per QALY gained through participation in the Mamanet programme remains well below internationally recognized willingness-to-pay thresholds (USD 50,000–100,000 per QALY ( (19, 34), indicating that the programme delivers good value for money and is cost-effective from a public funding perspective (36). Integrating such programmes into national health insurance reimbursement mechanisms may reduce systemic barriers and encourage sustained participation. Subsidized fees and local leagues ensure access for all women. Sustained efforts and inclusive strategies are key to promoting gender equity in sports and enabling all women to benefit from participation.

These frameworks provide decision-makers with data-driven evidence for the efficient allocation of resources and the promotion of a gender-inclusive sports infrastructure (37). While traditional health metrics assess physiological outcomes, QALYs allow valuation of well-being improvements resulting from enhanced self-esteem, reduced depression, and greater social engagement (38, 39). In the context of women's team sports, these qualitative gains often translate into significant functional improvements in work, parenting, and community roles. CBAs also allow for the inclusion of indirect economic gains. For example, Mamanet participants often report fewer sick days, increased work attendance, and greater job satisfaction (14). These productivity outcomes, while sometimes underemphasized in public health literature, represent a critical source of cost savings and economic value. Moreover, improved emotional regulation and reduced stress levels contribute to a more stable workforce, particularly for women balancing employment with family responsibilities.

Willingness-to-pay (WTP) models are also employed to estimate perceived value by participants. Studies indicate that women in structured team sport programmes often report that the social and psychological benefits they gain exceed the actual monetary cost of participation, suggesting strong consumer surplus. This finding reinforces the case for subsidized or publicly funded programmes for lower-income populations who may otherwise be excluded from participation (40).

Another economic implication is the reduced reliance on pharmacological and clinical mental health services. Participants engaging in sustained team sport involvement report decreased medication use, fewer doctor visits, and reduced hospitalization rates (12). These outcomes translate into measurable savings for national health insurance systems and reduce burdens on overwhelmed mental health services. From a system-wide perspective, when sports interventions are adopted at scale, the cumulative financial savings can be substantial. Preventive models like Mamanet offer longitudinal benefits: reduced prevalence of chronic disease, improved population mental health, enhanced civic participation, and intergenerational transmission of healthy behaviours (8). Public investment in such models yields not only financial returns but enhanced resilience of health and social systems. It is also important to highlight the role of sports in promoting social mobility and labour market advancement. Team sports, by building confidence, discipline, and social networks, improve women's chances for promotion, leadership, and re-entry into the workforce. These macroeconomic effects, although slower to materialize, contribute meaningfully to gender equity, productivity, and national competitiveness.

The evaluation of preventive health interventions must consider both direct and opportunity costs to ensure a comprehensive understanding of their economic implications. Direct costs refer to expenses directly associated with participation, including registration fees, travel expenses, and necessary equipment. These practical costs are essential for assessing the affordability and accessibility of programmes, particularly for economically disadvantaged populations. According to the Centers for Disease Control and Prevention (CDC), direct expenditures encompass staff, fringe benefits, consulting fees, supplies, travel, and other contractual costs. The National Institutes of Health (NIH) similarly defines direct costs as those attributable to specific institutional activities. These standards underscore the importance of including direct costs in the financial analysis of health programmes. The World Health Organization emphasizes the need for cost-effective approaches to achieve health equity (22).

Opportunity costs, by contrast, represent the value of time participants might otherwise dedicate to paid work or personal responsibilities. This includes time spent attending medical checkups, health workshops, or community-based fitness programmes. Recognizing these trade-offs is vital to understanding the full economic impact of preventive interventions. As Culyer and Chalkidou (41) note, economic costs reflect the lost opportunity to apply resources elsewhere. Eime et al. (15) show that opportunity costs associated with community sports are outweighed by their health and social returns.

## Methods

### Research design and sampling

This research utilized a quantitative cross-sectional design to assess health, psychological, and social outcomes among women participating in Mamanet programmes. A stratified sampling method was employed to capture socioeconomic and geographic diversity among Arab participants across geographic regions and socioeconomic levels as defined by the Israel Central Bureau of Statistics (42).

The present study focused exclusively on Arab women participating in the Mamanet programme, which operates within Israel's multicultural context. While Mamanet as a national league includes both Jewish and Arab teams, the current sample comprised Arab participants only. This subgroup was selected to examine the programme's health, social, and economic impacts within a population often facing greater structural and cultural barriers to physical activity. Although intergroup dynamics were not directly analyzed, the broader multicultural framework of Mamanet characterized by bilingual communication and inclusive community engagement remains an essential component of its design. Future research may extend these findings by comparing outcomes across different cultural groups participating in the league.

The Ethics Committee of the Faculty of Health Sciences approved this study. All respondents were given explanations before data collection and were advised that participation was voluntary. The number of the ethics committee is AU-HEA-RT-20221225.

The study included 174 newly recruited Mamanet mother-players. The subjects completed questionnaires twice: in November 2023 (pre-test) prior to the Mamanet intervention and in August 2024 (post-test) following 10 months of Mamanet participation. Arab subjects were recruited in collaboration with Mamanet. The organization provided contact details of team captains, who distributed the study questionnaire through their team WhatsApp groups, a primary communication channel used among league members. This non-random recruitment strategy ensured that recruits were active members of Mamanet and had direct access to the online study materials. The approach leveraged existing team-based social structures to facilitate engagement and data collection at pre-test and post-test. The questionnaires in Hebrew and Arabic were digitized by Sekernet^1^, uploaded to an online link and distributed via WhatsApp by the coaches. The link to the questionnaire was tested on a small group of recruits and then distributed to all subjects. The responses were transferred to Sekernet to assist the researchers in data collection.

### Cultural adaptations and inclusiveness procedures

The implementation of the Mamanet programme among Arab women incorporated several cultural and logistical adaptations designed to enhance accessibility and inclusion within Arab communities. All study materials and questionnaires were administered in Arabic through trusted local team captains. Participation costs were deliberately modest, and practice schedules were adapted to accommodate family and caregiving responsibilities. Activities were held in venues located within or near participants' residential areas to minimize travel burden and ensure social safety. These adaptations reflect evidence-based principles of culturally tailored physical activity interventions shown to increase adherence and perceived safety among women from minority or traditional societies (17, 43).

While Mamanet as a national league promotes intercultural inclusion between Jewish and Arab women, the current evaluation focused specifically on Arab participants, capturing the effectiveness of these culturally adapted components within that community.

### Data collection and measures

The structured questionnaire was developed, integrating validated instruments from the scientific literature. Specifically, self-rated health was measured using the single-item general health question adapted from the SF-36 Health Survey (Framework IC., 1992). Depressive and somatic symptoms were assessed using the Patient Health Questionnaire [PHQ-9 (44)]. Physical activity levels were measured using the International Physical Activity Questionnaire (IPAQ) short form (45). Social capital indicators, including trust, reciprocity, and civic participation, were adapted from the Short Social Capital Assessment Tool (46) and from Putnam's social capital framework (47). Dietary habits were measured using items from the Mediterranean Diet Adherence Screener [MEDAS; Martínez-González et al. (48)].

The survey included measures of self-reported health status, BMI, chronic disease presence, psychosomatic symptoms, depressive tendencies, and indicators of social capital (trust, social involvement, support networks). Key metrics included self-reported health status (rated 1–6), psychosomatic symptoms on a 4-point scale, and social engagement frequency.

Economic evaluation applied a cost-benefit framework, assessing improvements in QALYs, reductions in healthcare costs, and enhanced social capital. Costs considered included direct (registration, travel, equipment) and opportunity costs (time).

Health outcome variables included general health perception (1–6 scale, converted to utility scores), mental well-being (7-point Likert scale), psychosomatic symptoms (8-item symptom checklist), and physical activity (International Physical Activity Scale), with WHO guidelines used to categorize activity levels into utility scores.

Social capital and community engagement were measured via six items, including volunteering and participation in community events, rated on a 5-point scale and converted into utility values reflecting potential reductions in service reliance. Participants also reported comorbidities (e.g., asthma, arthritis, hypertension, diabetes, cancer), which were used to assess potential medication cost savings based on improvements in reported health status.

### Data analysis and economic evaluation

All main variables were inspected for normality using Q–Q plots. Most variables met normality assumptions and were therefore analyzed using parametric statistics. Descriptive statistics (means, standard deviations, and frequencies) were used to summarize sociodemographic and outcome variables. Paired-sample *t*-tests were conducted to compare pre- and post-test scores, and effect sizes were calculated using Cohen's *d* following Morris and DeShon (49).

The physical activity variable, however, displayed a skewed distribution and violated the assumption of normality. Accordingly, it was analyzed using the non-parametric Wilcoxon signed-rank test, with results presented as median and interquartile range (IQR) rather than mean and standard deviation.

A CBA assessed health gains (via QALYs), social engagement (via utility-based service savings), and economic impacts (e.g., reductions in doctor visits, sick days, and medication usage). Physician visits and sick day reductions were monetized using standard Israeli rates (e.g., 200 ILS per doctor visit; 300 ILS per sick day). Medication cost reductions were calculated by applying condition-specific savings rates to self-reported health improvements.

Total programme benefits were aggregated across QALY improvements, social capital effects, and economic savings. Costs were calculated as the sum of direct (125 ILS registration), opportunity (based on 1.25 weekly hours × 40 weeks × 30.6 ILS), and travel/equipment (200 ILS/month travel for 10 months, 500 ILS/year for gear). The BCR was computed as total benefits divided by total costs, with BCR > 1 indicating economic viability.

To predict changes in health status, binary logistic regression was conducted. Improvement was defined as moving from “very bad,” “not good,” or “OK” to “good,” “very good,” or “excellent” from pre- to post-test. Only variables significantly associated with improvement were included in the model. All statistical analyses were conducted using SPSS version 29, with significance set at *p* < .05 (2-tailed).

The detailed and precise methodology for calculating health and economic outcomes, as well as the specific variables used in this analysis, is provided in an Supplementary Appendix. This Supplementary File elaborates on the step-by-step procedure for assigning utility values to various health and social indicators, calculating QALYs and estimating the economic impact of participation in Mamanet. By grounding these calculations in health economics theory and incorporating standard cost-benefit methodologies, the data in the Supplementary Appendix ensures transparency and replicability in assessing the programme's impact.

## Results

### Sociodemographic characteristics of study participants

The study intervention included 174 subjects with a mean age of 43.1 + 5.7. Most participants were Arab-Muslim (75.3%, *n* = 131), in a relationship (86.2%, *n* = 150), and had either a professional certificate (32.2%, *n* = 56) or a high school education (28.1%, *n* = 49). Regarding employment, 54.0% (*n* = 94) were employed, 17.2% (*n* = 30) were self-employed, and 16.1% (*n* = 28) were identified as housewives. For additional information, refer to Table 1.

**Table 1:** Sociodemographic characteristics of study participants (*N* = 174).

Characteristic	Category	Mean (SD) or N (%)
Age, years		43.1 (5.7)
Family status	Single	14.0 (8.0)
	In a relationship	150.0 (86.2)
	Divorced	3.0 (1.7)
	Widowed	7.0 (4.0)
Children, number		3.1 (1.2)
Education	High school	49.0 (28.1)
	Professional certificate	56.0 (32.2)
	Bachelor’s degree	34.0 (19.5)
	Graduate degree	35.0 (20.1)
Religion	Arab – Muslim	131.0 (75.3)
	Arab – Christian	36.0 (20.6)
	Druze	3.0 (1.7)
	Other	5.0 (2.8)
Religiosity level	Secular	56.0 (32.1)
	Traditional	34.0 (19.5)
	Religious	54.0 (31.0)
	Orthodox	12.0 (6.9)
	Declined or unknown	18.0 (10.3)
Employment status	Employee	94.0 (54.0)
	Self-employed	30.0 (17.2)
	Unemployed	4.0 (2.3)
	Housewife	28.0 (16.1)
	Other	18.0 (10.3)

SD, standard deviation.

Key takeaway: The sample consisted entirely of Arab women, most of whom were married, employed, and had moderate education levels, reflecting the representative socioeconomic profile of Mamanet participants.

### Changes from pre- to post-test in health outcomes and behaviour

According to Table 2, a significant improvement in general health was observed: the percentage of participants reporting “very good” or “excellent” health increased from 59.2% (*n* = 103) at pre-test to 84.5% (*n* = 147) at post-test (*p* < 0.01). Psychosomatic symptoms significantly decreased from a mean score of 5.01 ± 4.41 at pre-test to 4.01 ± 4.02 at post-test (*p* = 0.01), with a small effect size (*d* = 0.23).

**Table 2:** Changes from pre- to post-test in health outcomes.

Variable	Pre-test	Post-test	Statistic t or Z (*p*-value) [effect size] or Chi-squared (*p*-value)
General Health: Very bad/Not good, *n* (%)	8.0 (4.6)	–	–
General Health: Ok/Good, *n* (%)	63.0 (36.2)	27.0 (15.5)	*χ*² = 9.82 (< 0.01)
General Health: Very good/Excellent, *n* (%)	103.0 (59.2)	147.0 (84.5)	χ² = 14.3 (< 0.01)
Mental well-being, mean (SD)	19.62 (4.39)	20.12 (3.47)	t = 1.68 (0.09) [0.12]
Psychosomatic symptoms, mean (SD)	5.01 (4.41)	4.01 (4.02)	t = −2.35 (0.01) [0.23]
Moderate-to-vigorous physical activity, median (IQR)	181.75 (61.00–243.00)	185.00 (120.00–300.00)	Z = −1.42 (0.15)

IQR, interquartile range; SD, standard deviation.

Key takeaway: Mamanet participation improved self-rated health and reduced psychosomatic symptoms, while mental well-being and physical activity levels showed smaller or non-significant changes.

Median moderate-to-vigorous physical activity (MVPA) showed a non-significant change, increasing from 181.75 min [IQR: 61.00–243.00] at pre-test to 195.00 min [IQR: 120.00–300.00] at post-test (*p* = 0.15, Wilcoxon signed-rank test), with a small effect size (*ES* = 0.28). No significant change was observed in mental well-being scores (*p* = 0.09), with a negligible effect size (*ES* = 0.12).

### Benefits calculation

#### Health outcomes—quality of life score

**Figure 1:**
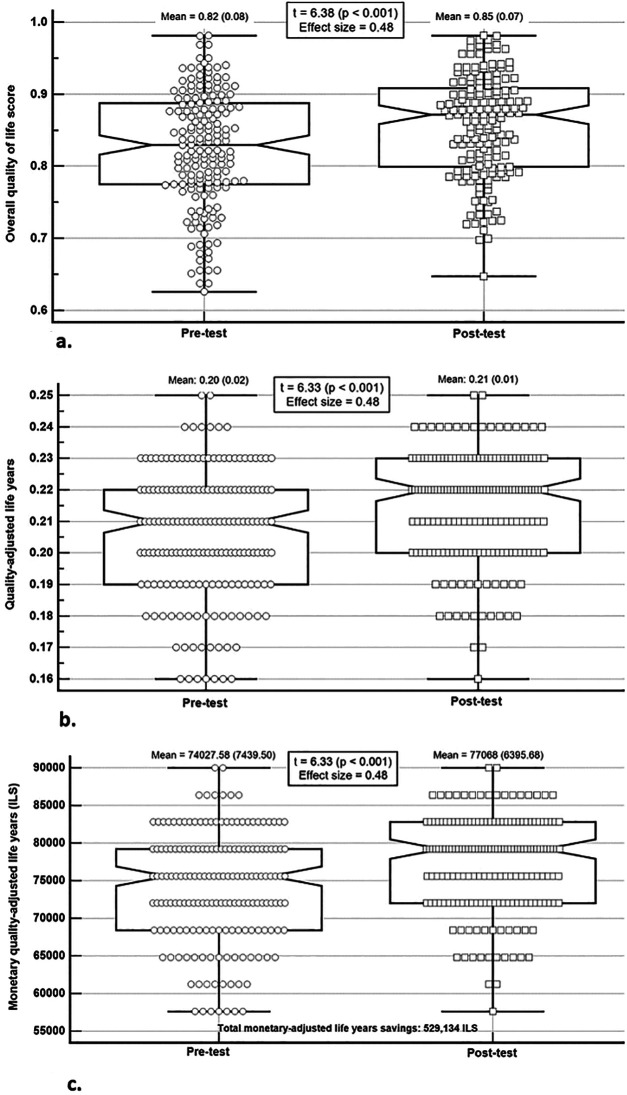
Changes in quality of life outcomes before and after Mamanet participation (N = 174). **(a)** Changes in overall quality of life. **(b)** Changes in adjusted year's quality of life. **(c)** Changes in monetary quality adjusted life years. Notes: The central box represents the values from the lower to upper quartile (25-75 percentiles); the vertical line extends from the minimum to the maximum value, excluding outside values which are displayed as separate points. An outside value is defined as a value that is smaller than the lower quartile minus 1.5 times the interquartile range, or larger than the upper quartile plus 1.5 times the interquartile range; the middle line represents the median. All measures demonstrated significant improvements between pre-test and post-test assessments (*p* < 0.001; ES = 0.48). Boxplots show median, interquartile range, and outside values.

The QAL score was calculated based on four key health domains: general health, mental well-being, psychosomatic symptoms, and PA. A significant improvement was observed in the overall quality of life score, increasing from 0.82 ± 0.08 at pre-test to 0.85 ± 0.07 at post-test (t = 6.38, *p* < 0.001), with a moderate ES = 0.48 (Figure 1A). Similarly, QALYs increased significantly from 0.20 ± 0.02 to 0.21 ± 0.01 (t = 6.33, *p* < 0.001), with a moderate ES = 0.48 (Figure 1B). Lastly, monetary QALYs showed a significant increase from 74,027.58 ± 7,439.50 ILS at pre-test to 77,068 ± 6,395.68 ILS at post-test (t = 6.33, *p* < 0.001), with a moderate ES = 0.48 (Figure 1C).

#### Social capital and community engagement

Social capital and community engagement were evaluated using a six-item questionnaire. Changes from pre- to post-test were calculated for each item, and a CBA. Regarding changes in social capital and community engagement from pre- to post-test, the results show a small but significant increase in participation in community events from 3.86 ± 1.41 to 3.98 ± 1.35 (*p* = 0.02, ES = 0.16), while all other indicators showed no significant changes. Accordingly, no significant changes were observed in the cost-benefit outcomes related to changes in social capital and community engagement from pre- to post-test. Nonetheless, the total cost benefit from changes in social capital and community engagement over the intervention period was 10,725 ILS across the entire sample (see Table 3).

**Table 3:** Changes from pre-test to post-test and cost benefit in social capital.

Variables	Pre-test mean (SD)	Post-test mean (SD)	t (p-value)	Effect size (d)	Pre-test cost (ILS)	Post-test cost (ILS)	Total cost benefit – ILS (post-test−pre-test)
**Changes from pre- to post-test, score**							
Participation in community events	3.86 (1.41)	3.98 (1.35)	2.22 (0.02)	0.16	–	–	–
Activity in local organizations and clubs	3.43 (1.51)	3.44 (1.50)	0.21 (0.83)	0.01	–	–	–
Participation in community projects	3.20 (1.54)	3.24 (1.56)	0.71 (0.47)	0.05	–	–	–
Volunteering	2.14 (1.56)	2.16 (1.59)	0.41 (0.67)	−0.03	–	–	–
Feeling appreciated by society	4.00 (1.36)	4.09 (1.37)	0.33 (0.73)	0.02	–	–	–
Receiving help from friends	3.55 (1.45)	3.64 (1.46)	2.16 (0.11)	0.16	–	–	–
**Cost benefit, Israeli Shekels**							
Participation in community events	390.58 (237.65)	400.00 (231.79)	0.87 (0.38)	0.06	66,400	68,000	1,600
Activity in local organizations and clubs	670.58 (474.11)	675.88 (478.88)	0.28 (0.71)	0.02	114,000	115,500	1,500
Participation in community projects	633.08 (527.26)	658.82 (541.91)	1.10 (0.27)	−0.08	112,000	107,625	4,375
Volunteering	319.52 (435.70)	329.88 (446.23)	0.54 (0.58)	0.04	54,000	55,750	1,750
Feeling appreciated by society	466.17 (255.40)	467.64 (255.95)	0.33 (0.74)	0.02	7,925	79,500	250
Receiving help from friends	421.32 (274.61)	428.67 (300.95)	0.74 (0.45)	−0.05	72,875	71,625	1,250
**Total**	–	–	–	–	–	–	10,725

Notes. ILS, Israeli Shekels.

Key takeaway. Although changes in social capital indicators were modest, a small yet positive total economic benefit (10,725 ILS) was observed, suggesting that community participation yields incremental but measurable social and financial gains.

#### Economic impact

**Figure 2:**
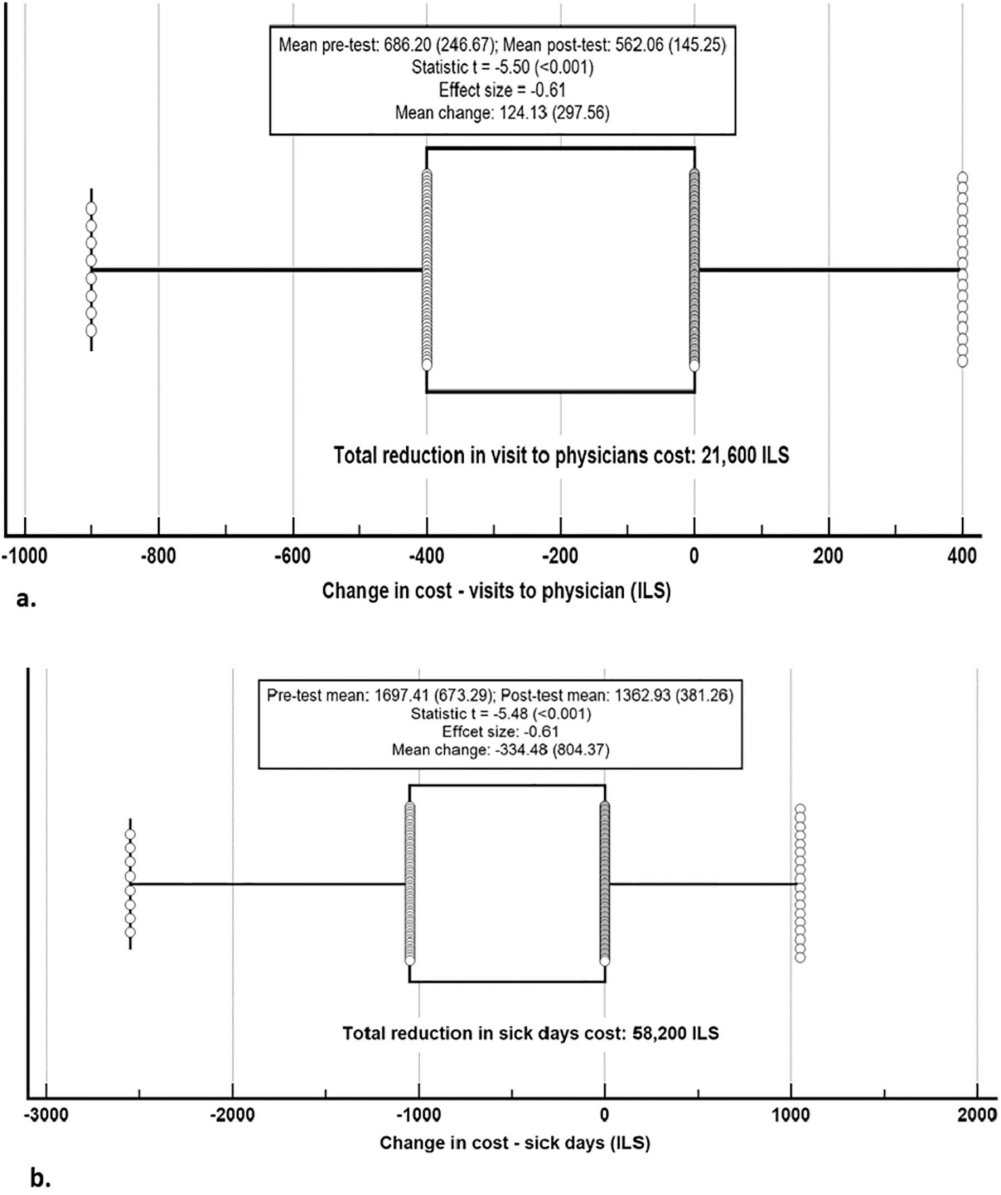
Changes in medical costs from pre-test to post-test. **(a)** Changes in visits to physician costs. **(b)** Changes in sick days costs. Notes: ILS, Israeli Shekel; Change cost calculation: pre-test cost - post-test cost. The central box represents the values from the lower to upper quartile (25-75 percentiles); the vertical line extends from the minimum to the maximum value, excluding outside values which are displayed as separate points. An outside value is defined as a value that is smaller than the lower quartile minus 1.5 times the interquartile range, or larger than the upper quartile plus 1.5 times the interquartile range; the middle line represents the median. Both categories demonstrated statistically significant cost reductionsf rom pre-test to post-test (*p* < 0.001). Costs expressed in ILS. Costs expressed in ILS. Boxplots indicate median, interquartile range, and outside values.

The economic impact was calculated based on changes from pre- to post-test in general health, and the estimated number and cost of physician visits, estimated number and cost of sick days, and medication costs for managing chronic health conditions. The results in Figure 2A show a significant reduction in the cost of physician visits, decreasing from a mean of 686.20 ± 246.67 ILS per person at pre-test to 562.06 ± 145.25 ILS per person at post-test (t = −5.50, *p* < 0.001, ES = −0.61). The average mean change per person was 124.13 ± 297.56 ILS, resulting in total savings of 21,600 ILS. Figure 2B demonstrates a significant reduction in sick day costs, decreasing from a mean of 1,697.41 ± 673.29 ILS per person at pre-test to 1,362.93 ± 381.26 ILS per person at post-test (t = −5.48, *p* < 0.001, ES = −0.61). The average reduction per person was 334.48 ± 804.37 ILS, leading to a total savings of 58,200 ILS.

As illustrated in Figuress 1,2, consistent improvements were observed in both quality-of-life indicators and healthcare-related economic outcomes.

To facilitate clearer interpretation of these results, Supplementary Appendix Table A1 provides a consolidated summary of the pre- and post-intervention changes presented in both figures, including corresponding *t* values, *p* values, and effect sizes.

Table 4 presents the estimated reduction in medication costs calculated for the 10-month intervention period among participants with chronic conditions whose general health status improved. The two medical conditions showing the highest cost reductions were diabetes, with total savings of 117,600 ILS, and arthritis, with 78,750 ILS. Together, these conditions contributed the most to the overall medication cost reduction, totaling 198,651.25 ILS. For example, 16 participants with diabetes improved their general health status. The mean monthly medication cost per patient was 4,200 ILS, corresponding to 42,000 ILS over 10 months. With a 17.5% reduction per improved participant, the savings per person amounted to 7,350 ILS over 10 months, resulting in 117,600 ILS in total savings for all improved diabetic participants. All cost calculations reflect the 10-month programme duration, not annualized values.

**Table 4:** Reduction in medication costs.

Health condition	Patients with the condition (N)	Patients improved^a^ (N)	Monthly medication cost (ILS)	10-month medication cost (ILS)	Reduction per improved patient (%)	Reduction per improved patient (ILS)	Total cost reduction for all improved patients (ILS)
Asthma	10	5	140	1,400	17.5	245	1,225
Arthritis	18	14	4,500	45,000	12.5	5,625	78,750
Hypertension	12	2	15	150	15	22.5	45
Clotting disorders	6	1	30	300	15	45	45
Anemia	1	–	–	–	–	–	–
Bleeding disorders	12	–	–	–	–	–	–
Osteoporosis	1	1	75	750	12.5	93.75	93.75
Thyroid dysfunction	2	1	30	300	10	30	30
Heart disease	9	5	115	1,150	15	172.5	862.5
Cancer	6	–	–	–	–	–	–
Kidney diseases	–	–	–	–	–	–	–
Liver diseases	–	–	–	–	–	–	–
Celiac disease	3	–	–	–	–	–	–
Parkinson	–	–	–	–	–	–	–
Stroke	1	–	–	–	–	–	–
Epilepsy	–	–	–	–	–	–	–
Diabetes	24	16	4,200	42,000	17.5	7,350	117,600
**Total**	–	–	–	–	–	–	**198,651.25**
–	**198,651.25**						

^a^ Improvement was defined as participants whose health changed from “Bad”, “Very bad”, or “OK” at pre-test to “Good”, “Very good”, or “Excellent” at post-test.

Notes. ILS, Israeli Shekels.

#### Total Mamanet benefits calculation

The total benefits of the Mamanet programme were calculated. The monetary value of QALYs improvement was 529,130 ILS (Figure 1C above). Additional benefits included a cost benefit from increased social capital and community engagement estimated at 10,725 ILS (Table 3), a reduction in physician visit costs of 21,600 ILS (Figure 2A), and a reduction in sick days cost of 58,200 ILS (Figure 2B above). Furthermore, the total reduction in medication costs for chronic conditions was 198,651.25 ILS (Table 4). Combined, the total estimated benefits of the intervention amounted to 818,306 ILS.

#### Costs calculation

The total cost of participating in the Mamanet programme was calculated by combining direct costs, opportunity costs, and travel and equipment expenses. Direct costs included programme registration fees of 125 ILS per participant, resulting in a total of 21,750 ILS for 174 participants. Opportunity costs were estimated based on the time commitment required for participation. With an average of 1.25 h per week, over 40 weeks at an average hourly wage of 30.6 ILS, the opportunity cost per participant was 1,530 ILS annually, amounting to a total of 266,220 ILS for 174 participants. Additionally travel expenses were estimated at 200 ILS per month over 10 months, totalling 2,000 ILS per participant annually, which amounts to 348,000 ILS for the entire group. Equipment costs were estimated at 500 ILS per participant per year, totalling 87,000 ILS for 174 participants. The combined total cost of the Mamanet programme for 174 participants was 722,970 ILS.

#### Benefit cost-ratio (BCR)

The BCR was calculated by dividing the total estimated benefits (818,306 ILS) by the total costs (722,970 ILS), resulting in a BCR of 1.13, indicating a positive economic return for every shekel invested.

#### Prediction of changes in health status

The logistic regression model predicting improvement in self-perceived health status was statistically significant (Chi-squared = 15.89, *p* < 0.01; Nagelkerke R^2^ = 0.12). Education level, age, and baseline PA were significant predictors of improvement. Participants with a bachelor's degree had significantly higher odds of reporting health improvement compared to those with a high school education (odds ratio = 1.35, 95% CI: 1.14–1.87, *p* = 0.02). Similarly, participants with a graduate degree were more likely to improve their health status (odds ratio = 1.38, 95% CI: 1.14–1.00, *p* = 0.05). Additionally, older age was associated with greater likelihood of improvement (odds ratio = 1.06, 95% CI: 1.00–1.13, *p* = 0.02). Finally, lower baseline levels of moderate-to-vigorous PA predicted improvement; as higher pre-test PA levels were negatively associated with improvement (odds ratio = 1.07, 95% CI: 1.01–1.14, *p* = 0.02). For additional information, see Table 5.

**Table 5:** Logistic regression for predicting improvement in self-perceived health status.

Variables	Coefficient	Standard error	Wald	*P*-value	Odds ratio (95% confidence interval)
Constant	3.16	1.32	5.67	**0** **.** **01**	–
Education—professional vs. high school	0.32	0.39	0.71	**0** **.** **39**	0.71 (0.33–1.54)
Education—bachelor’s degree vs. high school	1.02	0.45	5.09	**0** **.** **02**	1.35 (0.14–0.87)
Education—graduate school vs. high school	0.95	0.48	3.80	**0** **.** **05**	1.38 (0.14–1.00)
Age, years	0.06	0.02	5.13	**0** **.** **02**	1.06 (1.00–1.13)
Pre-test physical activity, minutes of moderate/vigorous activity	−0.07	0.01	5.14	**0** **.** **02**	1.07 (1.01–1.14)
Model summary	Chi-squared = 15.89 (*p* < 0.01); Nagelkerke R^2^ = 0.12				

Improved health status (*N* = 60) was defined as change from “very bad”, “not good” or “ok” in pre-test to “good”, “very good” or “excellent” at post-test; only independent variables that showed a significant difference in health status change were included in the regression model. The following variables were excluded: family status, religion, religiosity, employment, number of children, pre-test social capital, pre-test psychosomatic symptoms, pre-test mental well-being, and pre-test number of diseases. Bold values indicate statistically significant results (*p* < 0.05).

While cultural adaptations were not analyzed as separate predictors, the consistent pattern of improvements aligns with mechanisms described in culturally adapted PA programmes enhanced accessibility, social support, and sustained engagement (17, 43).

**Figure 3:**
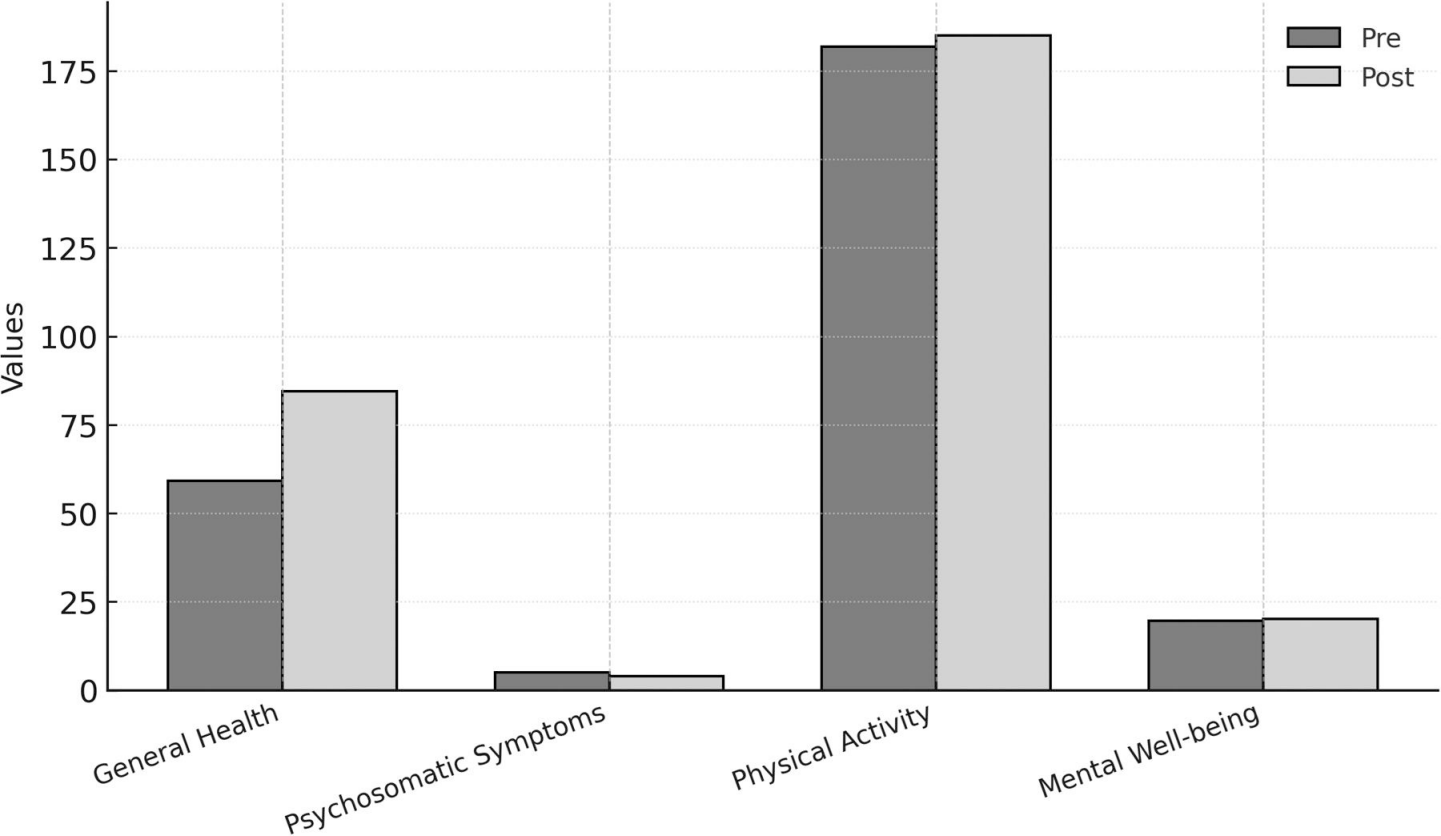
Outcomes at a glance.

To provide a concise overview of the multidimensional outcomes achieved, Figure 3 summarizes pre–post improvements across all measured indicators, including general health, psychosomatic symptoms, physical activity, and mental well-being.

## Discussion

The research results indicated significant improvements in overall physical health of Mamanet participants; the percentage of participants reporting “very good and excellent” health status increased from 59.2% to 84.5%. Psychosomatic symptoms showed a significant decrease (from 5.01 to 4.01), while mental well-being measures did not exhibit a statistically significant improvement (19.62 vs. 20.12, *p* = 0.09). Moderate-to-vigorous physical activity did not demonstrate a significant increase. The results confirm previous studies by demonstrating that consistent group PA enhances cardiovascular fitness, assists in weight management, and reduces the risk of chronic diseases (50).

The most significant decrease in medication expenses was noted among people with diabetes. This outcome was anticipated for multiple reasons. Diabetes emerged as the predominant chronic condition within the sample. Diabetes exhibits significant global prevalence, affecting nearly half a billion individuals. Projections indicate an increase of 25% by 2030% and 51% by 2045 (51). PA is essential for the prevention and management of diabetes (52, 53).

The decrease in psychosomatic complaints aligns with existing research indicating that organized exercise can alleviate stress-related and somatic symptoms by enhancing mood regulation and physiological resilience (52). However, the lack of substantial improvement in mental well-being scores underscores the complexity of quantifying psychological constructs such as anxiety, sadness, or self-esteem over short intervention periods (49, 54, 55). This pattern is consistent with previous studies showing that while physiological and somatic benefits of physical activity often emerge rapidly, measurable gains in subjective well-being typically require longer participation and broader psychosocial engagement (15, 16).

Overall, the consistent associations observed between participation and improvements in health and psychosomatic outcomes highlight the programme's practical relevance and potential effectiveness. Although the current design focuses on within-participant change rather than between-group comparison, it provides robust real-world evidence supporting the Mamanet model as a feasible and equitable health-promotion framework. Future comparative or longitudinal studies could extend these findings by elucidating the mechanisms underlying the observed associations.

Although no statistically significant improvement was detected in mental well-being (*p* = 0.09), this finding warrants careful interpretation. The result likely reflects both the limited duration of the 10-month intervention and the inherent difficulty of capturing subtle psychosocial changes through self-reported well-being scales. Prior research suggests that improvements in mental well-being resulting from structured physical activity often require longer participation periods and the accumulation of meaningful social experiences (15, 16).

Within culturally adapted community sports such as Mamanet, psychological benefits may unfold more gradually as participants develop stronger social bonds, trust, and a sense of belonging. These processes are linked to enhanced identity formation and perceived meaning key mediators of sustained psychological well-being (17, 43). Thus, while the current findings indicate short-term psychosomatic and physical gains, extended engagement and deeper cultural integration are likely necessary to achieve measurable improvements in mental well-being. Future longitudinal and mixed-methods research should further explore how inclusion, cultural adaptation, and team membership interact to promote resilience and sustained well-being among women participating in community-based sports initiatives.

Although the present analysis focused exclusively on Arab participants, Mamanet as a national model functions as a multicultural bridge, promoting intergroup contact between Jewish and Arab women through regional tournaments and community events. The current study did not include intergroup measures, which should be regarded as a limitation. Future research could explore how such cross-cultural interactions influence psychosocial well-being, identity formation, and community cohesion within Israel's diverse society.

Beyond these psychosocial outcomes, the economic evaluation further underscores the programme's societal value. The cost analysis revealed significant economic benefits with policy implications. For example, decreases in physician visit expenses, medication expenditures, and sick-day expenditures combined resulted in a favourable BCR of 1.13. Medication expenses associated with chronic diseases showed significant reductions, especially for diabetes and arthritis.

In CBA, the BCR offers a simple but powerful metric. A BCR exceeding 1.0 indicates that the programme's benefits outweigh its costs (36). Mamanet demonstrates this by improving health and reducing utilization of expensive medical interventions. The combination of improved health status, reduced healthcare spending, and increased productivity provides a strong justification for scaling the programme nationally and internationally.

From a policy perspective, these results underscore the need for integrating community-level sports into comprehensive public health initiatives. Research regularly demonstrates that preventative strategies particularly those targeting women can reduce the cost burden placed on healthcare systems (8). Legislators may contemplate: (a) specific funding for local sports facilities and leadership development, (b) incentive mechanisms such as reduced registration fees or insurance discounts, and (c) informational campaigns that emphasize the health and economic benefits of women's team sports (10, 56). Policymakers must develop criteria to assess the efficacy of team sports programmes in enhancing health and reducing healthcare costs, including both short-term and long-term indicators (54). Sustainability requires continuous funding, community engagement, and intersectoral collaboration to maintain access and effectiveness over time.

In the Mamanet case, direct costs such as registration, travel, and equipment are minimal compared to other organized sports, making the programme highly accessible. This affordability ensures that a broader population can participate, helping reduce disparities in health outcomes. The WHO emphasizes the need for cost-effective approaches to achieve health equity (1, 22).

For Mamanet participants, the time commitment required might replace other productive activities, such as employment. Nevertheless, these opportunity costs are offset by the substantial physical, mental, and social benefits that participants gain. The programme enhances personal well-being while cultivating community engagement, yielding long-term economic and societal value. By accounting for both direct and opportunity costs, stakeholders can better assess the full value of interventions like Mamanet, thereby supporting more effective allocation of public resources and informed policy decisions.

Mamanet stands as a replicable model for combining evidence-based public health with economic viability. Its success underscores the need to treat community-based sport programmes not as optional recreation, but as an integral public health infrastructure with lasting social and fiscal returns. By framing such interventions within health economics, policymakers and practitioners can advocate for broader, evidence-supported allocation of resources toward prevention and community resilience.

The recommendations for health promotion programmes and funding emphasize the significance of inclusive design, intersectoral collaboration, and a life-course approach. Inclusive design refers to the customization of women's sports schedules to accommodate caregiving responsibilities, professional commitments, and cultural factors (6). Such an approach involves offering shorter session formats, weekend leagues, and on-site childcare arrangements where appropriate, as well as ensuring subsidies or cost reductions when needed (57). Moreover, fostering intersectoral partnerships requires policymakers to coordinate with municipalities, healthcare providers, and schools to expand recruitment channels, lower logistical barriers, and promote broader demographic reach (58). This may involve municipal cooperation enabling community initiatives to utilize shared facilities, a healthcare framework where professionals encourage PA, and educational institutions that permit access for after-hours or weekend engagement (15).

Embracing a life-course perspective is a crucial element, especially considering the evidence that older individuals may derive health advantages from engaging in sports. By adapting sports to accommodate the abilities of older adults, teams can use lower-impact regulations and targeted coaching techniques, thereby enhancing chances for physical and social participation (59, 60). Facilitating diverse engagement throughout adulthood and midlife may substantially enhance long-term benefits for individual physical health and public health systems.

Practical implications for stakeholders are emphasized since intervention resulted in significant cost reductions (exceeding ILS 200,00 for particular chronic illnesses) in domains such as medications and medical consultations (61). This effect is pertinent to sports organizations, healthcare systems, and employers. Sports organizations should implement structured monitoring to continuously collect data on participants' health metrics, which can be essential to informing future funding and policy directives (62, 63). Emphasizing marketing and recruitment by sharing success stories and improved health outcomes is equally critical, as it may draw new participants, particularly those hesitant about their fitness levels or who have not previously engaged in such programmes. Healthcare systems play a decisive role by prescribing team sports involvement as a core element of preventive and rehabilitative care for chronic conditions (64, 65). These systems also stand to benefit from community partnerships that reduce hospital admissions or pharmaceutical expenses. Within this framework, they can collaborate with leagues like Mamanet to establish cost-sharing approaches aimed at decreasing overall medical utilization (66). Employers may integrate women's sports leagues into corporate wellness initiatives, either within the workplace or by sponsoring league fees. Flexible work schedules and compensated activity expenses are expected to enhance employee engagement, attendance, and productivity (67), thereby fostering an atmosphere where women's involvement in sports coincides with wider professional and familial responsibilities.

Community-driven sports initiatives like Mamanet may offer a support framework for women who may otherwise be marginalized in organized sports. Mamanet distinguishes itself by addressing participants' social and familial circumstances, hence enhancing adherence and increasing social capital. This methodology can serve as a template for other regions, given that it demonstrates how a sports model can be adapted to diverse cultural requirements. The notion of women deriving immediate health improvements translates into benefits for their families and communities at large, creating outcomes such as enhanced cohesion and reduced burdens on public health expenditures (36, 68). Furthermore, focusing on community engagement strengthens collective well-being and alleviates social isolation, hence fostering sustainable health habits over time (69). By fostering flexibility, inclusion, and mutual accountability within these community-based sports programmes, women may overcome conventional obstacles to participation, therefore gaining the extensive social and economic benefits associated with regular participation in organized PA.

The observed health and psychosocial improvements are consistent with evidence that culturally responsive, community-based sport initiatives generate both behavioral and social benefits. Bilingual delivery, trusted community gatekeepers, affordability, and proximity collectively lower entry barriers and strengthen commitment, while equal-status, goal-oriented teamwork supports cohesion and subjective well-being (17, 18, 25, 29). Although our design did not isolate these factors statistically, their integration into programme delivery plausibly contributed to the observed gains in participation, health, and social capital.

### Limitations

Notwithstanding the promising results, a few limitations require caution. First, the research assessed changes mostly across a single intervention cycle (about 10 months), which may not suffice to detect effects, particularly those related to mental health that may necessitate a longer period to become evident (70). Additionally, the study's reliance on general mental well-being metrics may inadequately reflect specific dimensions such as anxiety, sadness, or self-esteem. Other uncontrolled confounders such as diet, external stresses, and simultaneous health regimens may have influenced the findings. Furthermore, since participants in Mamanet are self-enrolled, this may suggest a level of motivation or health awareness not representative of the broader female population.

Considering these limitations, further research is warranted. Extending follow-up assessments at intervals such as 12, 18, and 24 months would allow for evaluation of the persistence of physical and psychological benefits. Expanding mental health indicators to include specific psychological dimensions (e.g., stress coping, self-esteem) could elucidate why certain mental health indicators improved while others remained unchanged. Future studies should also deepen demographic analyses to explore how socio-economic factors beyond age and education may mediate programme effectiveness or present barriers to participation (13). Incorporating qualitative research methods, such as focus groups or interviews, could further identify contextual facilitators and obstacles, particularly cultural or familial variables that may inhibit women's sustained engagement (13, 71).

The multicultural context pertains to the programme setting; the analytic sample comprised Arab participants only, and intercultural integration was not measured and therefore cannot be linked to outcomes in this study.

## Conclusion

This research emphasizes significant health improvements associated with organized, community-oriented team sports, as illustrated by the Mamanet Cachibol League in Israel. Mamanet participants showed diminished risk for chronic conditions such as hypertension and diabetes and reported enhanced psychosomatic well-being. While mental health outcomes varied, sustained involvement promoted better perceived health and quality of life. Beyond these improvements, the study identified economic advantages, including reduced healthcare utilization (fewer medications and physician visits) and increased productivity (fewer absences, more workplace engagement). The integration of CBA and QALYs methods yielded favourable BCRs, confirming that investments in women's team sports generate financial returns. These combined health and economic benefits support integrating community-based sports into public health policy.

Targeted efforts are recommended to expand women's participation, such as flexible scheduling, childcare support, and financial subsidies. Facility planning should ensure inclusiveness through dedicated spaces and culturally sensitive design. Community leadership is vital for sustainability. Including local women in planning ensures cultural relevance and fosters ownership. Encouraging leadership roles, such as team captains, strengthens empowerment and long-term engagement. Collaboration with health and education sectors is also recommended, for instance, encouraging physicians to prescribe sports for chronic disease prevention and using school facilities to instill lifelong activity habits.

Programme sustainability depends on embedding these initiatives within community structures that promote cohesion and support. Public and private partnerships should provide stable funding, subsidized fees, and accessible facilities, fostering inclusion across socioeconomic groups. The long-term success of these programmes is underpinned by their adaptability to diverse needs and family structures.

Future legislative initiatives should incorporate augmented financing for research on the long-term effects of team sports on public health. Further investigation is required to elucidate the processes by which team sports enhance physical, mental, and social well-being, and to assess their cost-effectiveness relative to alternative treatments. Financial resources should be allocated to the enhancement and proliferation of sports activities, especially in marginalized groups, by investing in infrastructure, such as community sports facilities and school programmes. These efforts must prioritize inclusion, guaranteeing that all individuals irrespective of age, gender, or socio-economic status can enjoy the benefits of sports.

Future research should broaden demographic representation across age groups, ethnicities, and geographic settings to increase generalizability. Further, using advanced psychological assessments can clarify how sports impact mental health, self-esteem, and social capital. Longitudinal studies are encouraged to examine sustained health and economic outcomes. Mixed methods approaches including interviews and focus groups can yield deeper insights into enablers and barriers to programme success. Comparative analyses between community-based sports and other public health approaches, such as personalized exercise regimen or digital fitness interventions, may also clarify their relative cost-effectiveness and potential for integration.

In conclusion, community-driven women's sports initiatives yield clear benefits, enhancing physical and mental health while delivering measurable economic gains. With inclusive design, cross-sector partnerships, and longitudinal research, such programmes can serve as enduring models for promoting women's public health. CBIs are an effective, sustainable strategy for improving public health. Initiatives like Mamanet demonstrate how culturally grounded, community-led models can reduce disparities and promote lasting, equitable health outcomes.

## Data Availability

The raw data supporting the conclusions of this article will be made available by the authors, without undue reservation.

## Footnotes

^1^Sekernet is an Israeli company that provides online survey services: Sekernet.co.il (in Hebrew).
