# Supplementary material for: From court to community: a cost–benefit evaluation of a community sports programme for Arab women in Israel's multiethnic context
Source: Front Glob Womens Health. 2025 Dec 11;6:1661221. doi: 10.3389/fgwh.2025.1661221 (PMC12738962; doi:10.3389/fgwh.2025.1661221)
Supplement: Supplementary file 1 [file Datasheet1.docx]

**Supplementary Material: Detailed Methods and Economic Evaluation Calculations**

**From Court to Community: A Cost-Benefit Evaluation of a Community Sports Program for Jewish and Arab Women in Israel’s Multiethnic Society**

Dr. Limor Dina Gonen, Ariel University, Israel Corresponding Author: limorg@ariel.ac.il

This appendix presents detailed procedures used to calculate health, social, and economic variables in the cost-benefit analysis of the Mamanet program. All calculations are grounded in health economics theory and aligned with peer-reviewed literature.

**Summary of Quantitative Results**
Consolidated summary table of key outcomes (*Appendix Table A1*), summarizing quality-of-life and healthcare-cost changes corresponding to Figures 1–2.

**This introductory summary table (Appendix Table A1) precedes the detailed methodological sections below and provides a concise overview of the key outcomes used in the subsequent analyses.**

1. Health Outcomes Analysis

- Assignment of utility values for general health, mental well-being, psychosomatic symptoms, and physical activity.
- Calculation of Quality of Life (QoL) scores and Quality-Adjusted Life Years (QALYs).
- Monetary valuation of QALYs using Israeli thresholds.

1. Social Capital and Community Engagement

- Assessment of participants' social trust, community involvement, and perceived social support.
- Calculation of financial savings from reduced reliance on social services, applying percentage reductions linked to community engagement levels.

1. Economic Impact and Healthcare Savings

- Estimation of reduced healthcare utilization: doctor visits, sick days, and medication costs.
- Application of health status-linked reductions and Israeli healthcare cost benchmarks.

1. Summary of Calculations

- Step-by-step methods for calculating total benefits, including QALYs, social capital, and healthcare savings.
- Formulae and example computations clearly presented.

1. Direct Cost Calculations

- Detailed breakdown of program fees, opportunity costs (time), and travel and equipment costs.
- Clear explanation of assumptions and data sources.

1. Benefit-Cost Ratio (BCR) Analysis

- Comprehensive calculation of BCR comparing total benefits to total costs.
- Interpretation of BCR results in the context of program viability.

1. References

- Full citation list of all supporting literature, including Israeli-specific sources.

This supplementary document ensures transparency, reproducibility, and rigor in evaluating the Mamanet program’s health and economic impacts.

**Summary of Quantitative Results**

# To improve transparency and interpretability of the statistical results, the following table consolidates the core numerical data underlying Figures 1 and 2 in the main text. It presents pre- and post-intervention values for key quality-of-life indicators and healthcare cost measures, including means, standard deviations, *t* values, *p* values, and effect sizes (Cohen’s *d*). This summary provides a concise reference for readers, facilitating clearer understanding of the magnitude and direction of change across physical, mental, and economic outcomes associated with Mamanet participation.

# **Table A1. Changes in Quality-of-Life Measures and healthcare costs from Pre- and Post-Intervention**

|  | **Measure** | **Timepoint** | **Mean**  **(standard deviation)** | **t value** | **p value** | **Effect size (Cohen’s d)** | **Mean Change (standard deviation)** | **Total Cost Reduction, Israeli Shekel** |
| --- | --- | --- | --- | --- | --- | --- | --- | --- |
| Quality of life measures | Overall quality of life score | Pre-test | 0.82 (0.08) | 6.38 | < 0.001 | 0.48 | - | - |
|  |  | Post-test | 0.85 (0.07) |  |  |  |  |  |
|  | Quality-adjusted life years | Pre-test | 0.20 (0.02) | 6.33 | < 0.001 | 0.48 | - | - |
|  |  | Post-test | 0.21 (0.01) |  |  |  |  |  |
|  | Monetary quality-adjusted life years, Israeli Shekel* | Pre-test | 74,027.58 (7,439.50) | 6.33 | < 0.001 | 0.48 | - | - |
|  |  | Post-test | 77,068.00 (6,395.68) |  |  |  |  |  |
| Healthcare costs | Cost – visits to physician, Israeli Shekel | Pre-test | 686.20 (246.67) | -5.50 | < 0.001 | -0.61 | 124.13 (297.56) | 21,600 |
|  |  | Post-test | 562.06 (145.25) |  |  |  |  |  |
|  | Cost – sick days, Israeli Shekel | Pre-test | 1697.41 (673.29) | -5.48 | < 0.001 | -0.61 | 334.48 (804.37) | 58,200 |
|  |  | Post-test | 1362.93 (381.26) |  |  |  |  |  |

Notes. Total monetary-adjusted life years savings: 529,134 ILS

**Notes.**
Values represent pre- and post-intervention means ± standard deviations. *t* values reflect paired-sample *t* tests; negative signs indicate post-intervention reductions. Effect sizes are reported as Cohen’s *d*.
Monetary values are expressed in Israeli shekels (ILS). Total monetary-adjusted life-year savings: 529,134 ILS.
This consolidated table supplements Figures 1 and 2, enabling direct comparison between health-related quality-of-life improvements and corresponding healthcare-cost reductions.

The following detailed explanation outlines the procedure for calculation of variables using data from the Mamanet questionnaire, grounded in health economics theory and supported by key literature in the field.

**1. Calculating Benefits**

1.1 Health Outcomes Analysis

This section aims to assess the impact of participation in Mamanet on self-reported health, chronic disease management, and mental well-being. It also evaluates how Mamanet participation influences psychosomatic and depressive symptoms. The analysis is conducted using utility values that measure participants' Quality of Life (QoL), which is further used to calculate Quality-Adjusted Life Years (QALYs). QALYs are an established metric in health economics to quantify the effectiveness of health interventions in terms of both the quality and quantity of life lived.

- 1. Key Research Questions:

1.2.1 How does participation in Mamanet impact self-reported health, chronic disease management, and mental well-being?1.2.2 What is the relationship between participation and improvements in psychosomatic and depressive symptoms?

1.3 Relevant Questionnaire Questions:

- General Health (question 36)
- Mental Well-Being (questions 37-40)
- Psychosomatic Symptoms (questions 41-49)
- Physical Activity (questions 51, 56, 59)

1.4 Steps for Analysis

1.4.1 Assign Utility Values

Utility values are assigned to participant responses to quantify health, mental well-being, and other related indicators. These values, ranging from 0 to 1, where 1 represents perfect health and 0 represents death, are used in the subsequent calculation of QoL and QALY.

1.4.2 Utility Assignment for General Health:

- Excellent → 1.0
- Very Good → 0.9
- Good → 0.8
- Fair → 0.6
- Poor → 0.4
- Very Poor → 0.2

This utility scale is grounded in health utility theory, where individuals' health statuses are rated and assigned values that reflect their overall well-being. These values can be used in health economics models to represent QoL in a way that allows for direct comparison across various health states and interventions (Gafni and Birch, 2006).

1.4.3 Utility Assignment for Mental Well-Being:

- 7 → 1.0
- 6 → 0.9
- 5 → 0.8
- 4 → 0.7
- 3 → 0.6
- 2 → 0.4
- 1 → 0.2

This scale is similarly used to measure well-being and is commonly applied in studies that involve quality-of-life assessments in mental health. For example, Hirth et al. (2000) examined various scales for assigning utility values to different health and mental states, showing the reliability of these methods for comparative analysis.

1.4.4 Utility Assignment for Psychosomatic Symptoms:

- 0 (Not at all) → 1.0
- 1 (Several days) → 0.75
- 2 (More than half the days) → 0.5
- 3 (Almost every day) → 0.25

Psychosomatic symptoms, such as headaches or fatigue, are measured on this scale, with greater frequency correlating to lower utility values. These scales are standard in cost-utility analysis and are supported by the literature, which suggests that more frequent symptoms reduce overall QoL (Hirth et al., 2000).

1.4.5 Utility Assignment for Physical Activity:

Physical activity is considered a proxy for physical health, with higher levels of activity associated with higher QoL values:

- Vigorous activity 5-7 days per week → 0.9–1.0
- Moderate activity 3-4 days per week → 0.7–0.8
- Low or no activity → 0.4–0.6

Engaging in frequent physical activity is often associated with improvements in both physical and mental health, leading to higher utility values in such analyses. This scale aligns with studies like that by Gafni and Birch (2006), which emphasize the use of physical activity as a direct indicator of health in economic evaluations.

1.4.6 Calculate Overall Quality of Life (QoL) Score

The overall QoL score is calculated by averaging the utility values assigned to the participant's responses across several categories (e.g., general health, mental well-being, physical activity). The formula used is:

- - Formula: $\frac{sum of utility value}{number of categories}$​

Example Calculation:

A participant rates their health across four dimensions:

- General health status: "Good" → Utility = 0.8
- Happiness: Rated 5 on a scale of 7 → Utility = 0.8
- Mental well-being: Rated 6 → Utility = 0.9
- Physical activity: Engages in moderate activity 4 days per week → Utility = 0.75

The overall QoL score is:

- - Overall QoL= $\frac{0.8+0.8+0.9+0.75}{4}$ =0.8125

This process is grounded in health economics literature, where QoL scores are calculated by aggregating various health-related utility values (Hirth et al. 2000, Gafni and Birch 2006).

1.4.7 Calculate QALY (Quality-Adjusted Life Years)

Once the overall QoL score is determined, the next step is to calculate QALYs.

A QALY is a measure of disease burden, including both the quality and quantity of life lived. It is calculated by multiplying the QoL score by the number of years the individual has lived or is expected to live at that level of QoL:

- - QALY=QoL score ×Years lived

Example Calculation:

If a participant’s QoL score is 0.8125 and the analysis is for a period of 1 year, the QALY is:

QALY=0.8125×1=0.8125

If the analysis spans multiple years, multiply the QoL score by the total number of years. Tracking changes over time allows for adjustments to the QALY if QoL improves or declines due to participation in Mamanet (Gafni and Birch, 2006).

1.4.8 Assign Monetary Value to QALY in Israel

In Israel, the value of one QALY is estimated to be 360,000 ILS (Hirth et al. 2000).

The monetary value of a participant’s QALY is calculated by multiplying the QALY by this figure:

Monetary Value=0.8125×360,000=292,500 ILS

Example Calculation:

For a participant with a QoL score of 0.8125 over 1 year, the monetary value of their QALY would be:

Monetary Value=0.8125×360,000=292,500 ILS

This method of calculating the monetary value of health outcomes is standard in cost-effectiveness analysis and is supported by studies such as that by Hirth et al. (2000).

**52. Social Capital and Community Engagement**

This section explores how participation in Mamanet impacts participants' social trust, community involvement, and perceived support, with a focus on how these factors affect emotional well-being and reduce social isolation. By measuring the reduction in reliance on social services and quantifying the financial savings, this analysis illustrates the broader socio-economic benefits of participation.

2.1 Key Research Questions:

2.1.1 How does participation in Mamanet impact social trust, community involvement, and perceived support?

2.1.2 What is the effect of participation on emotional well-being and social isolation?

Relevant Questionnaire Questions:

Participation in Community Events (question 72)

Activity in Local Organizations or Clubs (question 73)

Participation in Community Projects (question 74)

Volunteering (question 75)

Feeling Appreciated by Society (question 77)

Receiving Help from Friends (question 78)

2.2 Steps for Analysis

Assigning Utility Values Based on Participation

The analysis assigns different percentage reductions in reliance on social services based on the frequency of participation in community activities. The greater the engagement, the higher the reduction in social service reliance. The reduction percentages, ranging from 5% to 20%, reflect the positive impact of social involvement on well-being and social support networks.

2.3 Participation in Community Events (question 72):

1 (Never): No reduction in reliance on social services.

2 (Once or twice): Small reduction (~5%).

3 (Occasionally): Moderate reduction (~10%).

4 (Frequently): Significant reduction (~15%).

5 (Very frequently): Maximum reduction (~20%).

Example Calculation: If mental health services cost 2,000 ILS annually, a participant who frequently attends community events, resulting in a 15% reduction in social service reliance, would experience a benefit of: Benefit=0.15×2,000= 300 ILS annually.

This example demonstrates how frequent social participation can lead to substantial financial savings by reducing the need for formal mental health services. This method is supported by studies in community health and social capital that highlight the role of social engagement in reducing mental health service usage (Taub Center, 2020).

2.4 Mental Health Services and Cost Estimates

Private Therapy Costs:

In Israel, private therapy sessions range between 300 to 500 ILS per session, depending on the therapist’s qualifications and location. If a person attends therapy once a month (12 sessions per year), the annual cost would be:

300×12=3,600ILS annually (lower-end therapy)

For higher-end therapy, annual costs can reach up to 6,000 ILS ([Get Help Israel](https://gethelpisrael.com/psychiatric-services-in-israel-an-overview/), [Yad L’Olim](https://www.yadlolim.org/healthcare/mental-healthcare-guide)).

Subsidized Services (Kupot Cholim):

Many individuals in Israel receive subsidized mental health services through the public health system (Kupot Cholim), where therapy costs are reduced to around 100-200 ILS per session. For occasional therapy, this results in an average annual cost of approximately 2,000 ILS (Get Help Israel).

Online therapy services, such as those offered by platforms like [Mantra Care](https://mantracare.org/israel/online-therapy/), typically cost 280 to 360 ILS per session. These options are generally less expensive than in-person therapy and provide flexibility, reducing overall costs for individuals seeking mental health support ([Mantra Care](https://mantracare.org/israel/online-therapy/)).

Example Calculation (Subsidized Services):

For a participant using subsidized therapy (costing 100-200 ILS per session) and attending therapy once a month, the annual cost could range from 1,200 to 2,400 ILS. Therefore, a person who participates frequently in Mamanet, leading to a 15% reduction in service reliance, would save:

Benefit=0.15×2,000=300 ILS annually

This reduction in social service reliance is consistent with evidence showing that community engagement can improve mental well-being, reducing the need for formal therapy (Taub Center, 2020).

2.5 Activity in Local Organizations or Clubs (question 73)

Engagement in local organizations is linked to reduced reliance on formal social services. The analysis uses a similar percentage reduction model based on activity levels:

1 (Not at all): No reduction in reliance on social services.

2 (Slightly active): Minimal reduction (~5%).

3 (Moderately active): Moderate reduction (~10%).

4 (Very active): Higher reduction (~15%).

5 (Extremely active): Maximum reduction (~20%).

Example Calculation:

If a participant is very active in local clubs and reduces social service reliance by 15%, and if social services cost 3,000 ILS annually, the benefit would be:

Benefit=0.15×3,000=450 ILS annually

The cost of social services, such as social worker consultations, can range from 200 to 300 ILS per session, with annual costs between 2,000 and 4,500 ILS, depending on the frequency of use. This estimate is based on publicly available data from Bituach Leumi (National Insurance Institute of Israel) and other reports (Taub Center, 2020).

Private social worker consultations in Israel typically cost between 200 and 400 ILS per session depending on the provider. The cost for more intensive or specialized services could be higher. If an individual uses social worker services or community programs at an average rate of once a month, the annual expense would be around 3,000 to 4,800 ILS ([Study in Israel: How much does a social worker earn in Israel – in Hebrew](https://www.study.co.il/%D7%A9%D7%9B%D7%A8-%D7%A2%D7%91%D7%95%D7%93%D7%94-%D7%A1%D7%95%D7%A6%D7%99%D7%90%D7%9C%D7%99%D7%AA/?utm_source=chatgpt.com)).

2.6 Participation in Community Projects (question 74)

Participation in community projects strengthens social ties and reduces the need for formal social services:

1 (Never): No reduction in social service reliance.

2 (Once or twice): Small reduction (~5%).

3 (Occasionally): Moderate reduction (~10%).

4 (Frequently): Higher reduction (~15%).

5 (Very frequently): Maximum reduction (~20%).

Example Calculation:

For a person frequently participating in community projects, reducing social services by 15%, if annual social service costs are 3,500 ILS, the benefit would be:

Benefit=0.15×3,500=525 ILS annually

This figure is supported by cost estimates from the Taub Center (2019) and the Statistical Abstract of Israel (2023), which report that social service costs, including community support programmes, typically range from 3,000 to 4,500 ILS annually.

2.7 Volunteering (question 75)

Volunteering is linked to reductions in formal service reliance:

1 (Not at all): No reduction in reliance on formal services.

2 (Rarely): Small reduction (~5%).

3 (Occasionally): Moderate reduction (~10%).

4 (Frequently): Significant reduction (~15%).

5 (Very frequently): Maximum reduction (~20%).

Example Calculation:

If volunteering reduces social service reliance by 15%, and annual social service costs are 2,500 ILS, the benefit would be:

Benefit=0.15×2,500=375 ILS annually

This estimate is consistent with data from the Taub Center (2019) that outlines the costs associated with community involvement and social service reliance, from [Yahel Israel](https://www.yahelisrael.com/about-yahel-israelvolunteerprograms), which provides social change fellowship programmes that engage volunteers in community service and social work initiatives across Israel, and from [Go Overseas](https://www.gooverseas.com/) that outlines typical costs associated with volunteering in Israel, including programme fees and contributions for community involvement.

2.8 Feeling Appreciated by Society (question 77)

Feeling appreciated by society has a direct impact on mental well-being, which can reduce reliance on mental health services:

1 (Not at all): No reduction in reliance on mental health services.

2 (Rarely): Small reduction (~5%).

3 (Occasionally): Moderate reduction (~10%).

4 (Frequently): Significant reduction (~15%).

5 (Very frequently): Maximum reduction (~20%).

Example Calculation:

If mental health services cost 2,500 ILS annually, and feeling appreciated reduces service reliance by 10%, the benefit would be:

Benefit=0.10×2,500=250 ILS annually

This is supported by studies that show that increased social integration and mental well-being reduce the need for mental health services, leading to cost savings (Taub Center, 2020; [Get Help Israel](https://gethelpisrael.com/webpage/?title=therapy-through-kupat-cholim-), [Meuhedet](https://www.meuhedet.co.il/en/customer-information/mental-health-services/), [Therapy Helpers](https://therapyhelpers.com/blog/betterhelp-israel/).

2.9 Receiving Help from Friends (question 78)

Social support from friends can reduce the need for formal caregiving services:

1 (Not at all): No reduction in reliance on formal caregiving services.

2 (Rarely): Small reduction (~5%).

3 (Occasionally): Moderate reduction (~10%).

4 (Frequently): Higher reduction (~15%).

5 (Very frequently): Maximum reduction (~20%).

Example Calculation:

If formal caregiving services cost 4,000 ILS annually, and frequent help from friends reduces the need for such services by 15%, the benefit would be:

Benefit=0.15×4,000=600 ILS annually

The estimated cost of caregiving services ranges from 3,000 to 7,000 ILS per month, depending on the level of care needed. Occasional caregiving, with support from friends, reduces these costs to an estimated 4,000 ILS annually ([iSavta](https://www.isavta.co.il/en/work), Hasson and Buzaglo 2019, Taub Center 2020)

**3. Economic Impact and Healthcare Savings**

This section focuses on calculating the economic impact of Mamanet participation in terms of healthcare savings. The analysis assesses the reduction in healthcare utilization, including fewer doctor visits, medication costs, and sick days. These savings provide a framework for evaluating the cost-effectiveness of Mamanet participation and its impact on public health and economic well-being.

- 1. Key Research Questions:

3.1.1 What is the reduction in healthcare utilization (doctor visits, medications, sick days) among participants?

3.1.2 Relevant Questionnaire Questions:

- General Health (question 36)
- Medical History and Chronic Conditions (questions 14-30)

3.2 Steps for Analysis

- Reduction in Doctor Visits
- Identify Respondents with Good or Excellent Health

To assess healthcare savings, the analysis begins by identifying respondents who reported good or excellent health status in the questionnaire. Research shows that individuals in better health typically visit doctors less frequently, thus reducing healthcare costs.

3.2.1 Estimate Average Doctor Visits Based on Health Status

Participants reporting poor or fair health are estimated to visit the doctor 6-8 times per year, while those reporting good or excellent health visit only 2-3 times per year. These estimates are based on common healthcare utilization patterns in preventive health and chronic disease management (Ananthapavan et al., 2021).

3.2.2 Calculate the Benefit of Reduced Doctor Visits

To calculate the cost savings from reduced doctor visits, multiply the difference in doctor visits by the average cost per visit. In Israel, the cost of a general practitioner (GP) visit through private healthcare ranges from 250 to 1,000 ILS depending on the clinic, while visits through the public healthcare system (Kupat Holim) are often lower due to insurance coverage ([Yad L’Olim](https://www.yadlolim.org/healthcare)).

For this analysis, an average cost of 200 ILS per visit is used for simplicity.

Example Calculation: If individuals with good health have 3 fewer doctor visits per year compared to those with poor health, and the average cost per visit is 200 ILS, the savings would be:

Benefit from reduced doctor visits=3×200=600 ILS annually

This estimate is supported by research on cost-effectiveness in preventive health measures (van Boven et al. 2019, Hostetter et al. 2020, Rocks et al., 2020 ). Multiply this figure by the number of respondents reporting good or excellent health to estimate the total healthcare savings.

3.3 Reduction in Sick Days

3.3.1 Identify Respondents with Good or Excellent Health

Next, respondents who report good or excellent health are identified.

Research shows that healthier individuals take fewer sick days compared to those in poor health (Severin et al., 2022).

3.3.2 Estimate Sick Days Based on Health Status

Individuals with poor health may take 10-15 sick days per year, while those in good health take only 3-5 sick days. These estimates align with findings in workplace health promotion programs, where improved health status is linked to reduced absenteeism (Lutz et al., 2019).

3.3.3 Calculate the Benefit of Reduced Sick Days

To calculate the cost savings from fewer sick days, multiply the reduction in sick days by the average wage per day. The average wage in Israel is estimated at 300 ILS per day (Nefesh B’Nefesh, Decker Pex Levi [Law Office](https://lawoffice.org.il/en/sick-leave-in-israel/), [Kav LaOved-Workers Hotline](https://www.kavlaoved.org.il/en/full-time-workers-rights-sick-days/)).

Example Calculation: If individuals with good health take 5 fewer sick days per year, and the average wage per day is 300 ILS, the benefit from reduced sick days would be:

Benefit from reduced sick days=5×300=1,500 ILS annually.

This calculation is multiplied by the number of respondents reporting good or excellent health to estimate the total savings in lost wages.

3.4 Reduction in Medication Costs

3.4.1 Identify Respondents Diagnosed with Chronic Conditions

Respondents diagnosed with chronic conditions, such as asthma, arthritis, hypertension, and diabetes, are identified based on the relevant questionnaire questions (questions 14-30). These conditions often require long-term medication managem

3.4.2 Evaluate Management of Chronic Conditions

For each respondent, the analysis evaluates how well their condition is managed based on their reported general health. Those managing their condition well are expected to have lower medication costs.

3.4.3 Calculate the Benefit from Reduced Medication Costs

Medication costs vary based on the specific condition and the treatment regimen. The analysis estimates the average annual medication cost for each condition and calculates potential savings from better management of these conditions, resulting in fewer or less costly medications (Neumann et al. 2016).

Example Calculation: For hypertension, the average annual medication cost is estimated at 1,500 ILS.

If well-managed, medication costs could be reduced by 20%, resulting in savings of:

Benefit from reduced medication costs=0.20×1,500=300 ILS annually.

This calculation can be applied to other chronic conditions using similar logic. For example, asthma inhalers can cost between 80-200 ILS per month, and well-managed asthma could lead to a 15-20% reduction in costs (Yaghoubi et al. 2019, [The Pharmaceutical Society of Israel](https://drug.co.il/drugs/%D7%A1%D7%99%D7%9E%D7%91%D7%99%D7%A7%D7%95%D7%A8%D7%98-%D7%98%D7%95%D7%A8%D7%91%D7%95%D7%94%D7%9C%D7%A8-160-4-5-%D7%9E%D7%A7%D7%92-%D7%9E%D7%A0%D7%94/), [Shor Tabachnik medicine price list](https://shor.co.il/relvar-ell-184-22mcg-inh-%D7%A8%D7%9C%D7%91%D7%A8-%D7%9E%D7%A9%D7%90%D7%A3-184-22-%D7%9E%D7%A7%D7%92-%D7%9E%D7%A9%D7%90%D7%A3/?utm_source) – in Hebrew).

Medication Costs for Various Chronic Conditions:

1. Asthma: 960-2,400 ILS annually (80-200 ILS/month for inhalers). Potential savings: 15-20%.
2. Arthritis: 36,000-72,000 ILS annually (biologics). Potential savings: 10-15%.
3. Hypertension: 120-240 ILS annually (lisinopril). Potential savings: 10-20%.
4. Clotting disorders: 360-6,000 ILS annually (warfarin, newer anticoagulants). Potential savings: 15%.
5. Anemia: 240-1,200 ILS annually (iron supplements, erythropoiesis-stimulating agents). Potential savings: 10-15%.
6. Bleeding disorders: Tens of thousands of ILS annually (clotting factor replacements). Potential savings: 10%.
7. Osteoporosis: 600-2,400 ILS annually (bisphosphonates). Potential savings: 10-15%.
8. Thyroid dysfunction: 240-480 ILS annually (levothyroxine). Potential savings: 10%.
9. Heart disease: 360-2,400 ILS annually (statins, beta blockers, anticoagulants). Potential savings: 15%.
10. Cancer: Tens of thousands of ILS annually (biologics, chemotherapy). Covered by insurance.
11. Kidney diseases: 1,200-12,000 ILS annually (erythropoiesis-stimulating agents, phosphate binders). Potential savings: 10-15%.
12. Liver diseases: Ten of thousands of ILS annually (antivirals). Covered by insurance.
13. Parkinson's disease: 600-2,400 ILS annually (levodopa). Potential savings: 10-15%.
14. Stroke: 600-1,800 ILS annually (anticoagulants). Potential savings: 10%.
15. Epilepsy: 600-1,800 ILS annually (anticonvulsants). Potential savings: 10-15%.
16. Diabetes: 2,400-6,000 ILS annually (insulin). Potential savings: 15-20%.

These estimates reflect current drug costs in Israel’s healthcare system (Health Policy Journal, 2020; [Archives of Medical Science](https://www.archivesofmedicalscience.com/doi/10.5114/aoms.2020.92689)). The total benefit is calculated by multiplying the potential savings by the number of respondents with well-managed conditions.

Supporting Literature:

- Lomnicky et al. 2016.
- Arbelle et al. 2014.
- Neumann et al. 2020.
- Wierzejska et al. 2020.

Summary of Calculations:

1. Reduction in Doctor Visits: 600 ILS per year for participants in good health.
2. Reduction in Medication Costs: 300 ILS per year for each well-managed chronic condition.
3. Reduction in Sick Days: 1,500 ILS per year for participants in good health.

By applying these benefit calculations, the cost-benefit analysis model for Mamanet participation is developed. Each category—doctor visits, medication costs, and sick days—demonstrates tangible economic savings derived from improved health status and better disease management.

The total benefit in the context of the programme, as described in the document, is calculated by combining health, social, and economic outcomes.

**4. Health Outcomes**

Variables/Factors:

- General Health: Utility values are assigned based on self-reported health status (e.g., "Good" = 0.8 utility).
- Mental Well-being: Scored on a 1–7 scale, converted to utility values (e.g., 5 = 0.8 utility).
- Psychosomatic Symptoms: Frequency of symptoms reduces utility values (e.g., "Almost every day" = 0.25 utility).
- Physical Activity Frequency: Higher frequency correlates with higher quality of life (QoL).

Calculation:

- QoL Score: Average utility values across health-related responses.

QoL=Sum of Utility Values/Number of Questions

Example: QoL=0.8+0.8+0.9+0.75/4=0.8125

- QALY (Quality-Adjusted Life Year): Multiply the QoL score by the analysis period (e.g., 1 year):

QALY=QoL×Years

Example: QALY=0.8125×1=0.8125

- Monetary Value of QALY: Convert QALY to monetary terms using the national value (360,000 ILS per QALY):

Monetary Value=QALY×Value of 1 QALY

Example: Monetary Value=0.8125×360,000=292,500 ILS

**5. Social Capital and Community Engagement**

Variables/Factors:

- Participation in community events, local clubs, projects, and volunteering.
- Reduction in reliance on formal social or mental health services (5%-20% depending on activity level).

Calculation:

- Reduction in Service Costs: Benefit = Reduction Rate × Annual Cost of Services

Example: For frequent volunteering reducing reliance by 15%, and services costing 2,500 ILS annually:

Benefit = 0.15×2,500 = 375 ILS Sum benefits across all community engagement variables.

**6. Economic Impact and Healthcare Savings**

Variables/Factors:

- Reduction in doctor visits, sick days, and medication costs.
- Improved management of chronic conditions.

Calculation:

- Reduction in Doctor Visits:

Benefit = (Reduction in Visits) × (Cost per Visit)

Example: 3 fewer visits/year at 200 ILS per visit:

Benefit = 3 × 200 = 600 ILS/year

- Reduction in Sick Days:

Benefit = (Reduction in Sick Days) × (Daily Wage)

Example: 5 fewer sick days/year at 300 ILS/day:

Benefit = 5 × 300 = 1,500 ILS/year

- Reduction in Medication Costs:

Benefit = (Cost Reduction Rate) × (Annual Medication Cost)

Example: 20% reduction in 1,500 ILS annual cost for hypertension:

Benefit = 0.20×1,500 = 300 ILS

**7. Total Benefit Calculation**

Sum benefits across all categories:

Total Benefit = (Health Outcomes Monetary Value) + (Social Capital Benefits) + (Healthcare Savings)

**8. Calculating Costs**

This section focuses on calculating the direct costs associated with participation in the Mamanet programme. Mamanet is a sports league for mothers in Israel, and understanding the financial commitment involved helps determine the programme's economic viability. This analysis will examine the costs of participation, including programme fees, opportunity costs (time commitment), and travel and equipment costs. These direct costs are integral to calculating the overall benefit-cost ratio (BCR) of the programme, which will be explained at the end.

8.1 Direct Costs

Program Fees

The program fees are calculated by summing the registration fees for each participant in the Mamanet program. This cost represents a core part of the direct financial outlay.

Formula:

Total Registration Fees=Number of Participants×Fee per Participant

In the case of Mamanet, the registration fees typically range between 500 and 1,000 ILS annually per participant. These fees may vary depending on the location, facilities, and league-specific factors.

Example Calculation:

If there are 100 participants, and the annual registration fee is set at 750 ILS per participant, the total registration fees would be:

Total Registration Fees = 100 × 750 = 75,000ILS annually

This calculation is based on typical fee structures reported by programmes like the Kaplen JCC on the Palisades and CSIT, which offer similar community sports programmes.

8.2 Opportunity Costs (Time Commitment)

The opportunity cost of participation refers to the value of time that participants could have spent on other productive activities, such as paid work. This cost is calculated by estimating the hours spent in Mamanet activities and multiplying by the average wage rate in Israel.

Formula:

Opportunity Cost = Average Hours per Week × Weeks per Year × Hourly Wage

Average Hours per Week: Participants generally commit 2-4 hours per week to practices and games. For this calculation, we assume an average of 3 hours per week.

Weeks per Year: Mamanet activities typically run for about 40 weeks annually, accounting for holidays and breaks.

Hourly Wage: The average hourly wage in Israel is approximately 30.6 ILS, based on national labour statistics ([TimeCamp](https://www.timecamp.com/average-salary/israel/) 2024, [Expatistan](https://www.expatistan.com/cost-of-living/jerusalem)).

Example Calculation:

For a participant who commits 3 hours per week to Mamanet for 40 weeks, the opportunity cost would be:

Opportunity Cost = 3hours/week × 40weeks × 30.6ILS/hour = 3,672ILS annually

This value represents the forgone earnings or value of time participants could have spent on alternative productive activities. The opportunity cost is a key consideration in benefit-cost analyses of time-intensive programs like Mamanet ([Kaplen JCC on the Palisades](https://www.jccotp.org/programs/mamanet/), [CSIT](https://www.csit.sport/currently/news/mamanet-every-mother-can)).

8.3 Travel and Equipment Costs

Travel Costs

Participants may incur travel costs to attend weekly practices and games. These costs depend on the mode of transportation, the frequency of travel, and the distance to venues.

- Public Transportation: In Israel, the monthly cost for public transportation (e.g., buses and trains) ranges from 200 to 400 ILS, depending on the distance and frequency of travel ([Rothberg International School](https://overseas.huji.ac.il/student-life/preparing-for-and-managing-costs/); [Papaya Global](https://www.papayaglobal.com/countrypedia/country/israel/)).

Private Vehicle Use: If participants use their own cars, travel costs can increase, factoring in fuel, maintenance, and parking.

Example Calculation (Public Transportation):

Assuming an average monthly travel cost of 300 ILS, the annual travel cost for a participant using public transportation would be:

Annual Travel Cost = 300 ILS/month × 12months = 3,600 ILS annually

Equipment Costs

Participants also need to purchase sports equipment, such as sportswear, shoes, and any necessary gear. These equipment costs typically range from 500 to 1,000 ILS annually ([CPA Dray](https://www.cpa-dray.com/en/blog/income-tax-deductible-expenses/), [Kaplen JCC on the Palisades](https://www.jccotp.org/programs/mamanet/)), depending on the quality and type of equipment required for participation.

Example Calculation (Equipment):

Assuming the average participant spends 750 ILS on sports equipment, the total cost would be:

Equipment Cost = 750 ILS annually

Total Travel and Equipment Costs

To determine the total travel and equipment costs for a Mamanet participant, sum the annual travel and equipment expenses:

Example Calculation:

If the travel cost is 3,600 ILS and the equipment cost is 750 ILS, the total cost would be:

Total Travel and Equipment Costs=3,600ILS+750ILS=4,350ILS annually

This estimate aligns with data from sources such as Rothberg International School and Papaya Global, which provide insights into transportation and equipment costs in Israel.

8.4 Benefit-Cost Ratio (BCR) Calculation

Once the direct costs of Mamanet participation (programme fees, opportunity costs, travel, and equipment costs) are calculated, the Benefit-Cost Ratio (BCR) can be determined. The BCR compares the total benefits of the programme (such as improved health outcomes and reduced healthcare costs) with the total costs.

Formula:

BCR = Total Benefits/Total Costs

​BCR > 1: The programme’s benefits exceed its costs, indicating that it is economically viable.

BCR < 1: The costs outweigh the benefits, suggesting that the programme may not be viable from an economic perspective.

Example Calculation:

Assume the total annual benefits (e.g., from improved health outcomes and reduced healthcare utilization) are estimated at 10,000 ILS, while the total costs (including programme fees, opportunity costs, travel, and equipment costs) are calculated as 8,022 ILS:

BCR=10,000/8022=1.25

Since the BCR is greater than 1, this suggests that the benefits of Mamanet participation outweigh the costs, making the programme economically viable.

**Conclusion**

The direct costs associated with Mamanet participation include programme fees, opportunity costs related to time commitment, and travel and equipment expenses. The BCR calculation allows for a comparison between these costs and the benefits participants derive from the programme, particularly in terms of health and social outcomes. By ensuring that the benefits exceed the costs, the programme demonstrates its economic value to participants and society at large.

**References**

Ananthapavan J, Moodie M, Milat A et al. A cost–benefit analysis framework for preventive health interventions to aid decision-making in Australian governments. *Health Research Policy and Systems.* 2021;19:1-23.

Arbelle JE, Chodick E, Goldstein A et al. [Multiple chronic disorders - health care system’s modern challenge in the Maccabi Health Care System | *Israel Journal of Health Policy Research.*](https://ijhpr.biomedcentral.com/articles/10.1186/2045-4015-3-29) 2014;3:29.

Central Bureau of Statistics. *Welfare - Statistical Abstract of Israel 2023, no. 7.* https://www.cbs.gov.il/en/publications/Pages/2023/Welfare-Statistical-Abstract-of-Israel-2023-No-74.aspx

Gafni A, Birch S. Incremental cost-effectiveness ratios (ICERs): the silence of the lambda. *Social Science & Medicine.* 2006;62(9):2091-2100.‏ https://doi.org/10.1016/j.socscimed.2005.10.023

Hasson Y, Buzaglo ND. *The Care Deficit in Israel: What it Means and How it Can be Reduced.* Adva Center, 2019.

https://adva.org/wp-content/uploads/2019/03/Care-Deficit-EN.pdf.

Hirth RA, Chernew ME, Miller, E et al. Willingness to pay for a quality-adjusted life year: in search of a standard. *Medical Decision Making.* 2000;20(3):332-342.‏

<https://doi.org/10.1177/0272989x0002000310>.

Hostetter J, Schwarz N, Klug M et al. Primary care visits increase utilization of evidence-based preventative health measures. *BMC Family Practice.* 2020;21:1-10.

https://doi.org/10.1186/s12875-020-01216-8

Lomnicky Y, Kurnik D, Loebstein R, et al. Trends in annual drug expenditure - a 16 year perspective of a public healthcare maintenance organization. *Israel Journal of Health Policy Research.* 2016;15:37. doi: 10.1186/s13584-016-0096-1.

Lutz N, Taeymans J, Ballmer C et al. Cost-effectiveness and cost-benefit of worksite health promotion programs in Europe: a systematic review. *European Journal of Public Health.* 2019;29(3):540-546.

Neumann PJ, Sanders GD, Russell LB et al. (eds.) *Cost-effectiveness in Health and Medicine*. Oxford University Press, 2016.

Rocks S, Berntson D, Gil-Salmerón A et al. Cost and effects of integrated care: a systematic literature review and meta-analysis. *The European Journal of Health Economics.* 2020;21:1211-1221. https://doi.org/10.1007/s10198-020-01217-5.

Severin J, Svensson M, Akerstrom M. Cost–benefit evaluation of an organizational-level intervention program for decreasing sickness absence among public sector employees in Sweden. *International Journal of Environmental Research and Public Health.* 2022;19(5):2998. https://doi.org/10.3390/ijerph19052998.

Taub Center. *An Unprecedented year for Israel’s Social Welfare System*. 2019.

https://www.taubcenter.org.il/en/an-unprecedented-year-for-israels-social-welfare-system/

Taub Center. *A Picture of the Nation 2020*. <https://www.taubcenter.org.il/en/pictures/2020/>.

van Boven JF, van de Hei SJ, Sadatsafavi M. Making sense of cost-effectiveness analyses in respiratory medicine: a practical guide for non-health economists. *European Respiratory Journal.* 2019;53(3): 1801816.

https://doi.org/10.1183/13993003.01816-2018.

Wierzejska E, Giernaś B, Lipiak A et al. A global perspective on the costs of hypertension: a

systematic review. *Archives of Medical Science.* 2020;16(1):1078-1091.‏ <https://doi.org/10.5114/aoms.2020.92689>.

Yaghoubi M, Adibi A, Safari A et al. The projected economic and health burden of uncontrolled asthma in the United States. *American journal of respiratory and critical care medicine*. 2019;200(9):1102-1112. https://doi.org/10.1164/rccm.201901-0016oc.
